# Strategy to overcome a nirmatrelvir resistance mechanism in the SARS-CoV-2 nsp5 protease

**DOI:** 10.1126/sciadv.adv8875

**Published:** 2025-06-06

**Authors:** Grace Neilsen, Shuiyun Lan, Ryan L. Slack, Zachary C. Lorson, Andres Emanuelli Castaner, Rachel Lee, Kristin G. Edwards, Huanchun Zhang, Jasper Lee, William A. Cantara, Maria E. Cilento, Hongwang Zhang, Ramyani De, Franck Amblard, Philip R. Tedbury, Karen A. Kirby, Raymond F. Schinazi, Stefan G. Sarafianos

**Affiliations:** ^1^ Center for ViroScience and Cure, Laboratory of Biochemical Pharmacology, Department of Pediatrics, Emory University School of Medicine, Atlanta, GA 30322, USA.; ^2^ Children’s Healthcare of Atlanta, Atlanta, GA 30322, USA.

## Abstract

E166V in the severe acute respiratory syndrome coronavirus 2 (SARS-CoV-2) nsp5 protease confers strong resistance to the antiviral component of Paxlovid, nirmatrelvir (NIR), in passaging and clinical samples. In SARS-CoV-2 replicons, E166V drastically decreased Washington (WA1) but not Omicron (BA.1) fitness (20- versus 2-fold), suggesting a lower barrier to resistance in the BA.1 strain and consistent with observed differences in respective nsp5 dimerization affinities. Crystal structures reveal a steric clash between the rigid, bulky NIR *tert*-butyl group and the β-branched Val^166^, disrupting the covalent binding of NIR to the catalytic Cys^145^ and leading to high resistance in BA.1 and WA1 replicons. NIR-resistant replicons remained susceptible to GC376, which can still covalently bind Cys^145^ by avoiding a steric clash with Val^166^ through “wiggling and jiggling.” Hence, strategic flexibility is a strategy that will help design second-generation antivirals against NIR-resistant viruses.

## Teaser

Conformational flexibility is used to overcome nirmatrelvir resistance in the SARS-CoV-2 nsp5 protease.

## INTRODUCTION

In 2019, severe acute respiratory syndrome coronavirus 2 (SARS-CoV-2) emerged and began a rapid global spread, leading to the COVID-19 pandemic. As of January 2025, COVID-19 has caused over 770 million cases and over 7 million deaths worldwide (*1*). This pandemic has also seen the advent of mRNA-based vaccines, which were rapidly produced and distributed, protecting millions of people (*2*–*4*). However, vaccines are predominantly effective as preventative measures and do not typically help people who are already infected. In addition, as the virus mutates and novel variants emerge (*5*, *6*), vaccine efficacy toward these variants has declined (*7*, *8*). Consequently, effective clinical control of the pandemic requires antivirals to treat infection and complement current prevention measures.

**Fig. 1.**
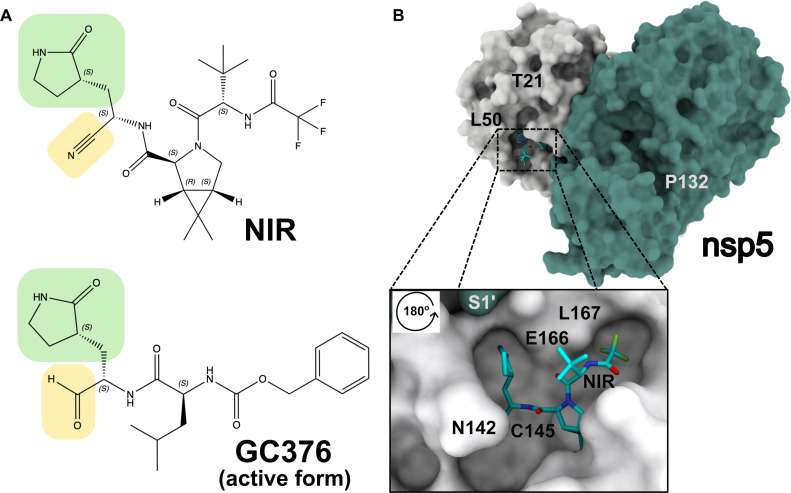
Nsp5 inhibitor structures and active site binding. (**A**) Structures of nsp5 inhibitors: nirmatrelvir (NIR) and GC376. Both compounds share a lactam ring (green) and a warhead (yellow) to form a covalent bond with Cys^145^. (**B**) Nsp5 dimer structure and close-up of one of the nsp5 active sites with covalently bound NIR (PDB ID: 7RFW). Nsp5 is shown as a light gray surface, and the N terminus of the other subunit (S1′) is shown as a teal surface.

The first Food and Drug Administration (FDA)–approved direct inhibitor of SARS-CoV-2 replication was remdesivir (RDV). RDV targets the viral RNA-dependent RNA polymerase or nonstructural protein (nsp) 12 (*9*). RDV was initially available in an injectable format, limiting its use to hospital settings; more recently, an orally available prodrug of RDV has been reported (*10*, *11*). Two additional drugs have been approved and can be used in oral formulations: Molnupiravir (*12*) and Paxlovid (*13*). Molnupiravir inhibits SARS-CoV-2 replication through viral RNA mutation buildup (*12*). A recent clinical trial reported that Molnupiravir treatment does not significantly lower the risk of hospital admission (*14*). Paxlovid targets the viral protease nsp5, also known as the main protease (M^pro^) or 3-chymotrypsin-like protease (3CL^pro^). Paxlovid is a combination of two drugs: nirmatrelvir (NIR) (Fig. 1A), a tripeptide-based antiviral that inhibits nsp5, and ritonavir, which improves the pharmacokinetic profile of NIR by inactivating the CYP3A4 enzyme. Nsp5 cleaves the viral polyproteins at 11 sites, releasing nsp4 through nsp16. It contains a catalytic dyad Cys^145^-His^41^ that bears similarities to that of chymotrypsin (*13*). GC376 also targets nsp5 (Fig. 1A). GC376 is a dipeptide-based broad-spectrum inhibitor of coronavirus nsp5 proteases with a bisulfite-aldehyde warhead, which reverts to the active aldehyde warhead under physiological conditions (also called GC373, the active GC376 conformation shown in Fig. 1A) (*15*). GC376 was initially discovered as an inhibitor of feline enteric coronavirus (FECV) and has now been shown to inhibit SARS-CoV-2 nsp5 (*15*–*18*).

NIR and GC376 mimic nsp5 substrates and covalently bind at the nsp5 active site through interactions between Cys^145^ and the inhibitor “warheads”: the cyano of NIR or the aldehyde of GC376 [derived under physiological conditions from the bisulfite group (*13*, *15*, *19*)]. Since receiving emergency use authorization (EUA) from the FDA on 21 December 2021, Paxlovid has been used to treat patients with COVID-19. Although several reports of SARS-CoV-2 rebound in Paxlovid-treated patients following treatment completion (*20*–*26*), these failures have not been linked to drug resistance mutations in nsp5. Recently, two clinical reports of virological rebound have linked Paxlovid failure to nsp5 mutations E166A/V (*25*) and L50V/E166V (*26*). These cases involved treatment using individual or combination therapies. Information on NIR resistance has also been obtained from virus passaging studies in cell culture experiments (*27*–*32*). These mutations often decreased viral replicative fitness and required compensatory mutations [such as T21I (*29*) and L50F (*28*, *29*)] to improve the fitness of the SARS-CoV-2 Washington (WA1) strain. Here, we predicted the NIR resistance effect of the E166V mutation based on analysis of nsp5 structures bound to NIR or natural substrates (*33*, *34*). We validated this hypothesis using virological data and provided the molecular mechanism for E166V-based NIR resistance using crystallographic and molecular dynamics (MD) simulation studies (*27*–*29*). We also describe the effects of NIR resistance mutations on replication fitness using cutting-edge replicon systems of Omicron (BA.1) and WA1 SARS-CoV-2 strains and in vitro using biochemical experiments. Last, we determine the effect of NIR resistance mutations on susceptibility to NIR and GC376 (Fig. 1A) (*35*). These data provide insight into molecular mechanisms of NIR resistance and a potential strategy to treat NIR (Paxlovid)–resistant SARS-CoV-2.

## RESULTS

### Impact of putative resistance mutations on the drug susceptibility and fitness of SARS-CoV-2 replicons

Using the crystal structures of WA1 nsp5, we designed mutations at residues proximal to the inhibitor binding site (Fig. 1B), two of which resulted in active proteases: N142L and E166V (fig. S1). To assess the effect of these residue changes on NIR resistance, we constructed mutant SARS-CoV-2 replicons derived from the WA1 and BA.1 Omicron strains. Like the corresponding viruses, the sequences of the WA1 and BA.1 replicons differ at 13 positions throughout the nsp genes, only one of which is in the nsp5 region (Pro^132^ in WA1 versus His^132^ in BA.1), specifically nsp3: K38R, S1265del, L1266I, and A1892T; nsp4: T492I; nsp5: P132H; nsp6: L105del, S106del, G107del, I189V, and L260F; nsp12: P323L; and nsp14: I42V.

#### Effect on fitness

**Fig. 2.**
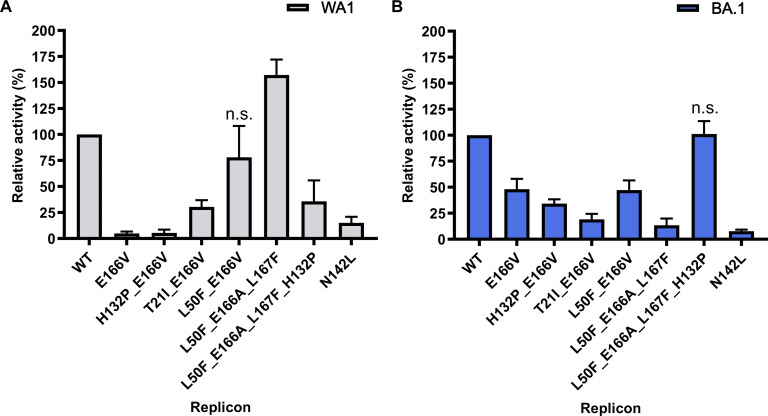
Impact of nsp5 mutations on replicon fitness of SARS-CoV-2 replicons. Relative replication fitness of WT and mutant replicons in WA1 (**A**) and BA.1 (**B**) backbones. *P* values determined by Dunnett’s multiple comparison test; all conditions had *P* < 0.0001 compared to WT except those indicated as not significant (n.s.). Error bars indicate SDs (*n* = 3).

To assess the replication fitness of the 16 replicons, we compared the reporter gene expression to that of the corresponding wild-type (WT) in the absence of antivirals when starting with equal amounts of bacmid DNA. N142L_WA1_ and E166V_WA1_ exhibited replication defects (~5-fold and over 10-fold, respectively) compared to WT_WA1_ (Fig. 2A). The previously reported L50F/E166A/L167F_WA1_ (*27*) replicated efficiently compared to WT_WA1_. Similar to previous reports (*28*, *29*), we found that introducing L50F into the WA1 E166V replicon (L50F/E166V_WA1_) restores fitness to WT levels in WT_WA1_, and introducing T21I (T21I/E166V_WA1_) partially restores fitness (Fig. 2A). However, in the BA.1 backbone, the E166V mutation did not decrease the fitness as much as it did in WA1 (E166V_BA.1_ had ~50% less activity of WT_BA.1_, compared to ~95% loss of activity of E166V_WA1_ compared to WT_WA1_), and the L50F and T21I mutations did not increase the fitness of E166V_BA.1_ (Fig. 2B). Notably, in contrast to the WA1 strain, the L50F/E166A/L167F_BA.1_ triple mutant appeared to have significantly decreased fitness compared to WT_BA.1_. To determine whether the effect of the triple mutation on fitness was the result of the single mutation in nsp5 residue 132, we flipped the P132H mutation in the triple mutant background. We found that H132P in BA.1 (L50F/E166A/L167F/H132P_BA.1_) rescued the poor fitness of the triple mutant (L50F/E166A/L167F_BA.1_). The effect was mirrored in WA1 where introducing P132H (L50F/E166A/L167F/P132H_WA1_) suppressed the strong fitness of the triple mutant (L50F/E166A/L167F_WA1_) (Fig. 2). However, P132H/E166V_WA1_ did not significantly increase the fitness, nor did H132P/E166V_BA.1_ show a significant decrease in fitness.

#### Effect on susceptibility to antivirals

We tested the antiviral susceptibility of 16 replicons, including several bearing putative nsp5 drug resistance mutations, to RDV, NIR, and GC376 (Table 1). RDV targets nsp12 and serves as a control. Hence, as expected, all replicons (WT_WA1_, WT_BA.1_, and all mutants in Table 1) had comparable median effective concentration (EC_50_) values for RDV. In contrast, the WA1 mutants displayed varying degrees of resistance to NIR with E166V_WA1_, T21I/E166V_WA1_, and L50F/E166V_WA1_ conferring the most resistance (131-fold, 250-fold, and 115-fold, respectively) and L50F/E166A/L167F_WA1_ and N142L_WA1_ conferring less resistance. GC376 displayed similar activity against all the mutants, with only L50F/E166A/L167F_WA1_ showing a small increase in EC_50_ (3.4-fold). The WT_BA.1_ was slightly more resistant than WT_WA1_ to all antivirals. Nonetheless, similar resistance patterns were seen in the BA.1 replicons with E166V_BA.1_, L50F/E166V_BA.1_, and T21I/E166V_BA.1_ showing comparable resistance to NIR and L50F/E166A/L167F_BA.1_ and N142L_BA.1_ showing less resistance to NIR. Again, only the L50F/E166A/L167F_BA.1_ (containing E166A, rather than E166V) demonstrated modest resistance to GC376 (Table 1).

**Table 1.** Susceptibility of WA1 (Washington strain) and BA.1 (Omicron strain) SARS-CoV-2 replicons to nsp5 inhibitors RDV, NIR, and GC376. RDV is an nsp12-targeting antiviral serving as a control. ND, not determined. Values represent average and SDs from *n* = 3 replicates. Bolded values in parentheses indicate fold change in EC_50_ compared to the respective WT.

	EC_50_ ± SD / μM (fold change from respective WT)		
nsp5 variant	RDV	NIR	GC376
WT_WA1_	0.010 ± 0.003 **(1)**	0.034 ± 0.009 **(1)**	0.21 ± 0.03 **(1)**
E166V_WA1_	0.011 ± 0.002 **(1.1)**	4.5 ± 1.1 **(131)**	0.19 ± 0.02 **(0.9)**
P132H/E166V_WA1_	ND	3.5 ± 0.7 **(102)**	ND
T21I/E166V_WA1_	ND	8.5 ± 1.7 **(250)**	ND
L50F/E166V_WA1_	0.007 ± 0.003 **(0.7)**	3.9 ± 1.1 **(115)**	0.49 ± 0.13 **(2.3)**
L50F/E166A/L167F_WA1_	0.011 ± 0.001 **(1.1)**	0.61 ± 0.05 **(18)**	0.71 ± 0.03 **(3.4)**
L50F/E166A/L167F/P132H_WA1_	ND	0.92 ± 0.3 **(27)**	ND
N142L_WA1_	0.009 ± 0.001 **(0.9)**	0.084 ± 0.01**(2.5)**	0.32 ± 0.03 **(1.5)**
WT_BA.1_	0.014 ± 0.002 **(1)**	0.11 ± 0.02 **(1)**	0.70 ± 0.24 **(1)**
E166V_BA.1_	0.006 ± 0.001**(0.4)**	7.3 ± 1.7 **(66)**	0.41 ± 0.20 **(0.6)**
H132P/E166V_BA.1_	ND	9.9 ± 1.4 **(90)**	ND
T21I/E166V_BA.1_	ND	12.0 ± 1.2 **(109)**	ND
L50F/E166V_BA.1_	0.006 ± 0.001 **(0.4)**	8.7 ± 0.5 **(79)**	0.62 ± 0.12 **(0.9)**
L50F/E166A/L167F_BA.1_	0.009 ± 0.004 **(0.7)**	2.9 ± 0.5 **(26)**	3.8 ± 2.0 **(5.4)**
L50F/E166A/L167F/H132P_BA.1_	ND	4.8 ± 2.7 **(44)**	ND
N142L_BA.1_	0.010 ± 0.001 **(0.7)**	0.11 ± 0.02 **(1.0)**	0.46 ± 0.14 **(0.7)**

### Resistance to nsp5 inhibitors in enzymatic protease activity assays

Median inhibitory concentration (IC_50_) values derived from an in vitro assay reflect this same pattern of resistance and susceptibility. An in vitro activity assay was used to determine IC_50_ values for the WA1 and BA.1 WT-nsp5 and E166V-nsp5 proteins with GC376 or NIR (table S1). This assay uses a fluorescently labeled peptide of the nsp4-5 cleavage site to measure nsp5 activity. As in the EC_50_ measurements, E166V-nsp5 proved more resistant to NIR compared to WT-nsp5 for both strains (9-fold for E166V-nsp5_WA1_ and 17-fold for E166V-nsp5_BA.1_) (table S1). However, GC376 still inhibits E166V-nsp5_WA1_ and E166V-nsp5_BA.1_ with similar IC_50_ values as the respective WT-nsp5 proteins.

### Analysis of viral sequences submitted in the GISAID database

Examination of the SARS-CoV-2 sequences in the Global Initiative on Sharing All Influenza Data (GISAID) h-Cov-19 database showed that all the examined mutations (Table 2) have a very low prevalence, most likely as a result of their impaired fitness. Almost all instances of E166V in the database occurred between the two time points. However, L50F [and T21I (*29*)] occurred at a considerably higher prevalence at both time points (>200-fold more than either mutation at Glu^166^) (Table 2) (*28*, *29*). In addition, a quarter of the sequences with the E166V mutations also contained the L50F mutation (13/46), and almost all (44/46) were from Omicron strains (i.e., contained P132H) (Table 2).

**Table 2.** Number of instances of each amino acid change as reported in the GISAID database. Values are instances reported of the 7,313,022 sequences (December 2021) and 15,634,584 (January 2025) analyzed.

Amino acid change	Instances December 2021	Instances January 2025
T21I	12,548	19,483
L50F	3,956	5,246
N142L	15	15
E166A	3	10
E166V	1	46
P132H/E166V	0	44
L50F/E166V	0	13

### Effect of mutations and drug binding on nsp5 stability measured by nanoDSF

Nano-differential scanning fluorimetry (nanoDSF) measurements provide insight into protein stability. For WT-nsp5_BA.1_ and E166V-nsp5_BA.1_ recombinant proteins, these data consistently showed that the melting curves for all proteins have similar inflection temperatures (*T*_i_), indicating no significant change in protein stability under these conditions (fig. S2 and table S2). In the presence of NIR, there was a significant increase in *T*_i_ of WT-nsp5_BA.1_ (from 58.4° to 75.6°C). However, there were only marginal changes in *T*_i_ in the presence and absence of NIR with E166V-nsp5_BA.1_ mutant protein complexes (Table 2). In contrast, the addition of GC376 caused significant shifts in *T*_i_ for both WT-nsp5_BA.1_ and E166V-nsp5_BA.1_ proteins. Similar results were obtained for WT-nsp5_WA1_ and E166V-nsp5_WA1_ (fig. S2 and table S2). Notably, although the presence of GC376 caused a change in *T*_i_ for all proteins, it was increased for the WT over the mutant proteins in both strains (up to twofold). These findings are consistent with the resistance profiles in the EC_50_ replicon assay (Table 1).

### X-ray crystallographic studies of E166V nsp5:inhibitor complexes

To study the effects of E166V on the structural interactions of nsp5 with the inhibitors, we solved the crystal structures of E166V-nsp5_BA.1_ in complex with either NIR or GC376 and compared them to existing WT-nsp5_BA.1_ complexes (*36*, *37*).

#### Mechanism of resistance

**Fig. 3.**
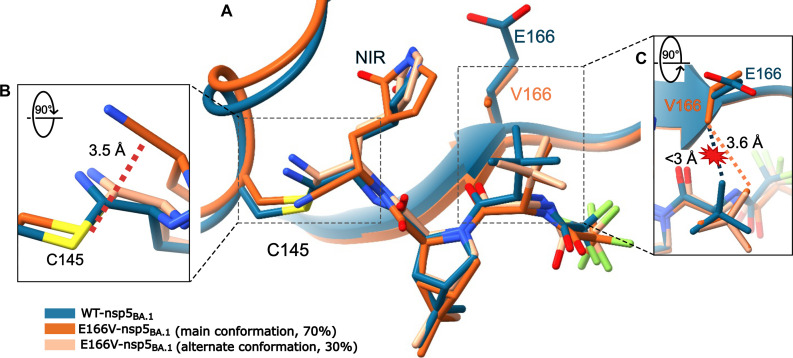
Steric hindrance from E166V weakens NIR binding. The β-branched V166 sterically interacts with the *tert*-butyl of NIR forcing it to reposition in the E166V-nsp5_BA.1_ active site. (**A**) Structure of E166V-nsp5_BA.1_ (orange) in complex with NIR compared to WT-nsp5_BA.1_ (blue; PDB ID: 7TLL). The alternate binding mode of NIR with a lower occupancy is shown in tan. (**B**) Rotated view of the catalytic C145 showing the increased distance between the C145_Sγ_ and the carbon of the nitrile warhead of the non-cross-linked binding mode (3.3 Å, which is incompatible with covalent binding). (**C**) Rotated view of the putative steric clash caused by the V166_Cγ_1__ with the NIR *tert*-butyl group, forcing NIR to reposition.

The 2.4-Å crystal structure of E166V-nsp5_BA.1_:NIR shows evidence of NIR entering the active site and assuming two slightly different binding modes (Fig. 3A). In one binding mode, NIR is capable of forming a covalent bond with the sulfur of the catalytic Cys^145^, although at a low apparent occupancy of ~30%. However, the other binding mode of NIR in the active site is incompatible with the formation of a covalent bond between Cys^145^ and the inhibitor and has an occupancy of ~70% (Fig. 3B). In addition, the β-branched Val^166^ is protruding into the active site, thus displacing the *tert*-butyl group of NIR in the P3 position from its position in the WT-nsp5_BA.1_:NIR complex to avoid a steric clash (Fig. 3C). There is no strong electron density around the warhead due to the multiple positions nor for the NIR trifluoro group at the opposite end of the molecule. These data support the hypothesis that E166V substantially decreases the covalent interactions of NIR with the E166V-nsp5_BA.1_ active site and catalytic Cys^145^.

#### Mechanism of overcoming resistance

**Fig. 4.**
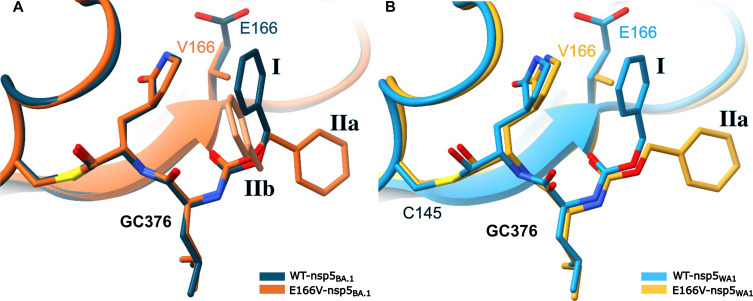
GC376 can avoid steric hindrance from E166V through flexibility. GC376 changes conformation in the E166V-nsp5_BA.1_ and E166V-nsp5_WA1_ active sites. (**A**) The benzyl group of GC376 adopts two different conformations (**IIa** and **IIb**; conformation **IIb** shown in light orange) when in complex with E166V-nsp5_BA.1_ (orange), both of which point away from V166 and do not align with the GC376 conformation (**I**) seen in WT-nsp5_BA.1_ (dark blue; PDB ID: 7TOB). (**B**) In E166V-nsp5_WA1_ (yellow), GC376 adopts a conformation similar to **IIa** in E166V-nsp5_BA.1_ (A). Aligned with WT-nsp5_WA1_ (light blue; PDB ID: 7JSU).

The E166V-nsp5_BA.1_:GC376 complex shows clear electron density bridging Cys^145^ and GC376 through a covalent bond (fig. S3), consistent with the observed efficient inhibition of the NIR-resistant E166V-nsp5_BA.1_ replicon by GC376. The conformation of GC376 generally resembles that seen in the WT-nsp5_BA.1_:GC376 [Protein Data Bank (PDB) ID: 7TOB (*37*)] with the notable exception of the benzyl ring (Fig. 4A). This moiety is pointed toward Glu^166^ in the WT-nsp5_BA.1_:GC376 complex (conformation **I** in Fig. 4). However, in the E166V-nsp5_BA.1_:GC376 complex, the benzyl ring assumes two conformations (best refined with occupancies ~45 and ~55%, respectively), both of which pointed away from Val^166^ (conformations **IIa** and **IIb** in Fig. 4A). Similarly, refinement of E166V-nsp5_WA1_ in complex with GC376 shows the benzyl ring exclusively in conformation **IIa** seen in the E166V-nsp5_BA.1_:GC376 complex (Fig. 4B and fig. S4).

#### Effect of the E166V mutation on nsp5 dimerization

The E166V mutation appears to also affect the architecture of the nsp5 active site by altering the intersubunit interactions involving the N terminus. In WT structures, Ser^1^′ of the neighboring nsp5 protomer forms hydrogen bonds with both the side chain of Glu^166^ and the main chain of Phe^140^ (fig. S5). However, in all E166V structures reported here, the N terminus changes orientation as Val^166^ cannot form a hydrogen bond with Ser^1^′. These changes may also contribute to the relatively weak density observed at the neighboring 140 to 146 region in the NIR structure without a covalently bound inhibitor or correctly placed N terminus to stabilize this loop at the S1 pocket.

### Disruption of NIR binding to nsp5 by E166V measured using BLI

**Fig. 5.**
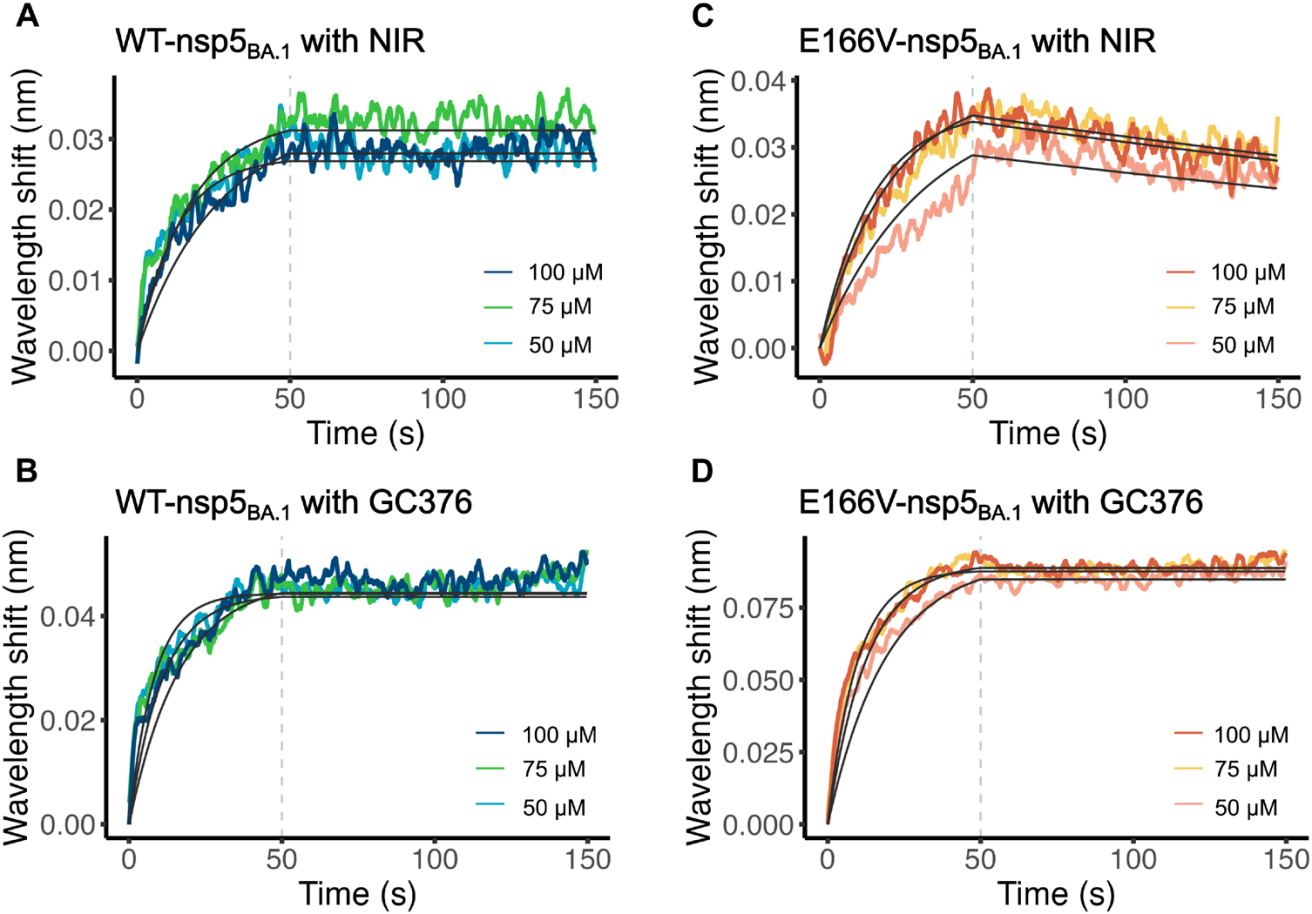
Covalent and noncovalent binding of nsp5 proteins to inhibitors. Covalent binding of NIR to WT-nsp5_BA.1_ (**A**) but not to E166V-nsp5_BA.1_ (**B**). Covalent binding of GC376 to WT-nsp5_BA.1_ (**C**) and E166V-nsp5_BA.1_ (**D**). Experiments were performed in triplicate at each of the three different inhibitor concentrations (50, 75, and 100 μM).

To obtain further insight into the mechanism of E166V-based NIR resistance, we used biolayer interferometry (BLI) experiments that provided biophysical insight into the binding affinities of NIR and GC376 to nsp5 proteins. For the WT-nsp5_BA.1_ protein, both NIR and GC376 bound with similar nanomolar binding affinities and did not dissociate from the protein over the time course of this experiment (Fig. 5, A and B, and Table 3), consistent with the observed formation of a covalent bond between the inhibitor and the catalytic Cys^145^ (Fig. 4). In contrast, E166V-nsp5_BA.1_ only showed this tight binding with GC376, not NIR (Fig. 5, C and D). While the on-rates (*k*_on_) are similar for WT-nsp5_BA.1_ and E166V-nsp5_BA.1_, the difference in affinity [dissociation constant (*K*_d_)] results from the significant decrease in off-rates (*k*_off_) for E166V-nsp5_BA.1_ with NIR (Table 3). These data demonstrated that NIR can noncovalently bind to E166V-nsp5_BA.1_ with similar kinetics as WT-nsp5_BA.1_ but cannot form a stable covalent bond like with WT-nsp5_BA.1_, consistent with crystal structures of the respective complexes (Figs. 3 and 4).

**Table 3.** Binding affinity (*K*_d_) and on-rate (*k*_on_) and off-rate (*k*_off_) parameters determined by BLI. Values represent average and SDs from *n* = 3 replicates. In the case of covalent binding, an upper limit is given for *k*_off_ and consequently *K*_d_.

	Drug	*K*_d_ / nM	*k*_on_ / M^−1^ s^−1^	*k*_off_ / s^−1^
WT-nsp5_BA.1_	+ NIR	<1.26	774 ± 9	<9.77 × 10^−7^
	+ GC376	<0.80	1220 ± 10	<9.77 × 10^−7^
E166V-nsp5_BA.1_	+ NIR	3370 ± 60	562 ± 5	1.89 ± 0.03 × 10^−3^
	+ GC376	<0.93	1050 ± 6	<9.77 × 10^−7^

### Effect of mutations and drug binding on nsp5 dimer-monomer equilibrium based on SEC-MALS

**Fig. 6.**
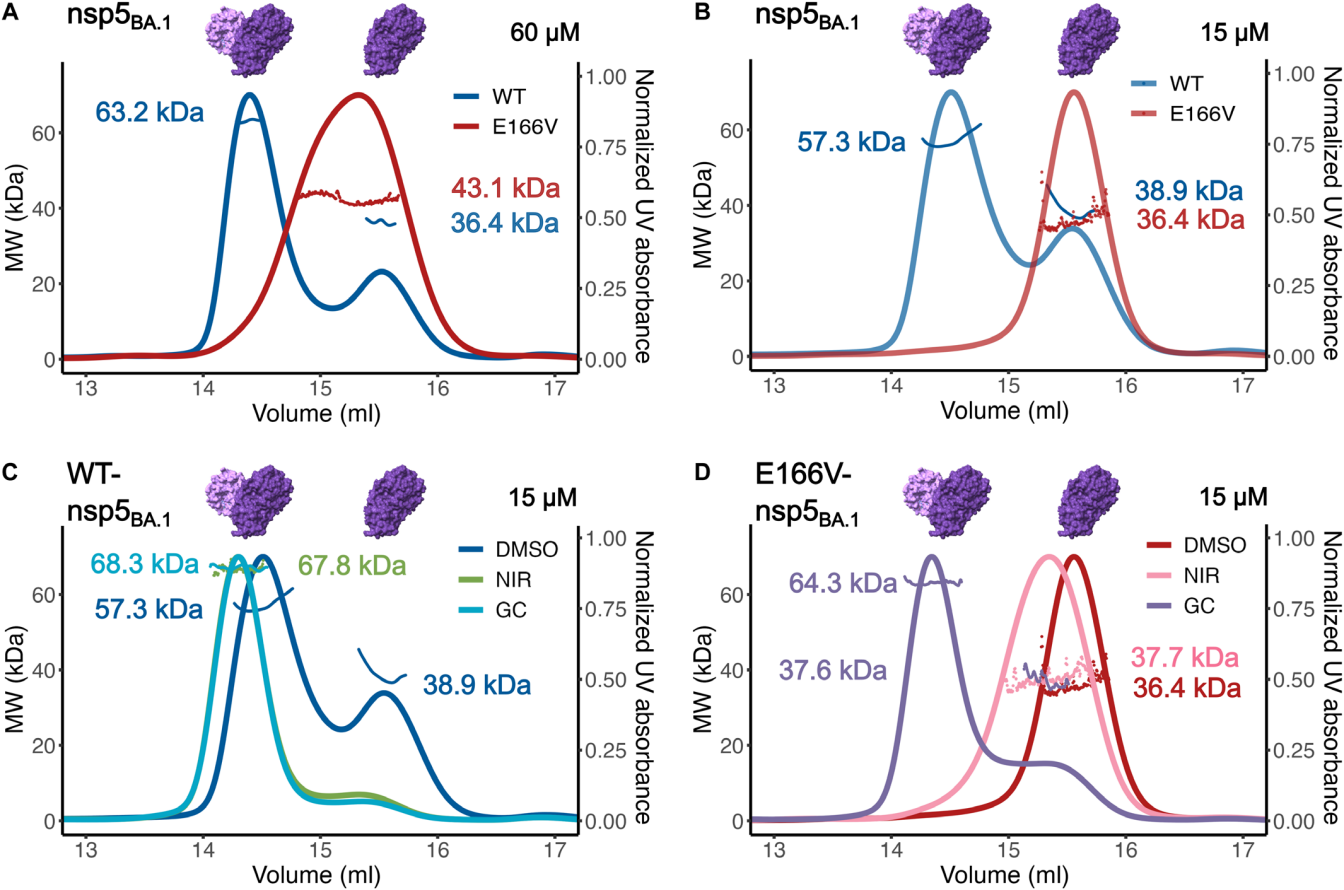
Effect of E166V on nsp5 dimerization. E166V decreases the dimerization of nsp5. SEC-MALS with WT-nsp5_BA.1_ and E166V-nsp5_BA.1_ proteins at 60 μM (**A**) and 15 μM (**B**). (**C**) WT-nsp5_BA.1_ (15 μM) in the presence of DMSO, NIR, or GC376. (**D**) E166V-nsp5_BA.1_ (15 μM) in the presence of DMSO, NIR, or GC376. Points indicate the molecular weight (MW) calculated based on the light scattering and UV absorbance (normalized UV absorbance shown as a trace). The expected MWs are ~33.8 kDa for a pure monomeric population and ~67.6 kDa for a pure dimeric population.

Because nsp5 is enzymatically active only as a dimer, studying the monomer-dimer equilibrium is essential for understanding the full effect of mutations and inhibitors on nsp5 activity. Normally, the N terminus of one protomer (Ser^1^′) stabilizes the S1 pocket in the active site of the opposite protomer and connects the dimer interface with the active site (fig. S5). In this pocket, Glu^166^ makes critical interactions with Ser^1^′ to help stabilize both the S1 pocket and the nsp5 dimer (*38*–*40*). A previous work with SARS-CoV nsp5 has suggested that mutations at Glu^166^ decreased dimer formation and protease activity (*38*). To understand the effect of E166V on nsp5 dimerization alone and in complex with inhibitors, we used size exclusion chromatography coupled to multiangle light scattering (SEC-MALS). These experiments revealed that E166V does perturb the monomer-dimer equilibrium of nsp5 proteins (Fig. 6). Whereas WT-nsp5_BA.1_ eluted as primarily dimeric with a small portion as a monomer, E166V-nsp5_BA.1_ eluted as a broad peak (Fig. 6A). This broad peak has an observed molar mass between the pure dimer and monomer values (~43 kDa compared to 67.6 kDa for dimeric nsp5 and 33.8 kDa for monomeric nsp5), possibly indicating a mixture of dimeric and monomeric species (table S3). When protein concentration was decreased, WT-nsp5_BA.1_ maintained a similar elution profile as primarily dimeric with an overlapping monomeric peak, and E166V-nsp5_BA.1_ proteins shifted to an exclusively monomeric state (Fig. 6B and table S3). Similar results were seen using nsp5_WA1_ proteins (fig. S6, A and B). In addition, a larger proportion of the WT-nsp5_BA.1_ remained dimeric at the lower concentration compared to the WT-nsp5_WA1_ (table S3). This difference in inherent dimerization efficiency could account for the different replication efficiency of E166V_WA1_ and E166V_BA.1_ replicons.

This technique also revealed how differences in inhibitor binding dynamics altered inhibitor-induced nsp5 dimerization in solution. Substrate and inhibitor binding has previously been linked to induced dimerization of nsp5 (*38*). SEC-MALS on nsp5 protein:inhibitor complexes provided insight into the different impact of covalent or noncovalent binding of inhibitors on the dimerization of WT-nsp5_BA.1_ and E166V-nsp5_BA.1_. Both NIR and GC376 result in equal shifts of WT-nsp5_BA.1_ to a fully dimeric state (Fig. 6C and table S3). In contrast, incubating E166V-nsp5_BA.1_ with NIR or GC376 led to varied results. With GC376, E166V-nsp5_BA.1_ markedly shifted to a mostly dimeric population, similar to WT-nsp5_BA.1_. However, incubating E166V-nsp5_BA.1_ with NIR, shown to not form a stable covalent complex, does not result in a similar shift. Instead, the E166V-nsp5_BA.1_ elution peak broadened and slightly increased in apparent molecular mass compared to the dimethyl sulfoxide (DMSO) control, indicating a mixture of the monomer and dimer species in the presence of NIR (Fig. 6D and table S3). Experiments with nsp5_WA1_ proteins yielded similar results with only NIR unable to shift E166V-nsp5_WA1_ to a dimeric state (fig. S6, C and D).

### MD analysis of nsp5 inhibitor binding

To further integrate the crystallographic, virological, and biochemical data, we conducted MD simulations of multiple nsp5-inhibitor complexes. The canonical active form of nsp5 is a dimer (*41*), which, at physiological conditions (pH ~ 7.4), has each substrate binding site positioned proximal to the N-terminal domain of the neighboring subunit. We initially ran pilot MD simulations of either the individual nsp5 monomers or dimers and did not observe significant structural differences between the active sites. Hence, we proceeded to study the following complexes in the dimer form, using simulations that were at least 100 ns in duration: WT-nsp5_WA1_:NIR, WT-nsp5_BA.1_:NIR, WT-nsp5_WA1_:GC376, WT-nsp5_BA.1_:GC376, E166V-nsp5_WA1_:NIR, E166V-nsp5_BA.1_:NIR, E166V-nsp5_WA1_:GC376, and E166V-nsp5_BA.1_:GC376. These simulations addressed the following points.

#### E166V confers resistance to NIR

During the simulations of the WT-nsp5_WA1_:NIR and E166V-nsp5_WA1_:NIR complexes, NIR moved as a rigid body and resulted in critical changes in conformation near the catalytic center. The inhibitors remained bound to the proteins and were generally well constrained throughout the simulation. NIR sampled a slightly narrower range of conformations in the WT-nsp5_WA1_ than in the E166V-nsp5_WA1_ complex (fig. S7A): Specifically, the average changes in displacement of non-hydrogen NIR atoms had root mean square deviation (RMSD) values of 1.2 Å versus 2.3 Å for the WT-nsp5_WA1_:NIR and E166V-nsp5_WA1_:NIR complexes. In addition, inspection of the individual NIR atom positions (fig. S7B) revealed overall minor changes in individual atom positions [typically ~1-Å root mean square fluctuation (RMSF)]. Apparent exceptions include the RMSF peaks at (i) carbon atoms in the *tert*-butyl (*t*-butyl) group and (ii) fluorine atoms in the trifluoromethyl group [fig. S7C, atoms (i) #16, #17, and #18; and (ii) #35, #36, and #37]. However, these changes reflect the sampling of identical rotamers that result from free rotation between the #21-#23 and #5-#22 chemical bonds (red and green circular arrows in fig. S7C). As such, these conformations are equivalent and equally present in both the WT-nsp5_WA1_ and E166V-nsp5_WA1_ simulations (fig. S7, B and C). We also observed minor changes at atoms near the Cys^145^-His^41^ catalytic center, namely, at the cyano group (#24 and #2) and at the atoms of the lactam ring (#29, #1, #9, #6, #7, #25, and #9) (fig. S7, B and C). Overall, NIR moves primarily as a rigid body during the simulations (fig. S7, D to F), albeit with a local torsional change (red arrow in fig. S7B) in the E166V-nsp5_WA1_ simulation. This torsional change results in a significant increase in the interatomic distances between the catalytic Cys^145^_Sγ_ and the reactive cyano carbon of the warhead [C2 in (C)] during the simulations of the E166V-nsp5_WA1_ versus the WT-nsp5_WA1_ NIR complex (6.2 Å versus 3.5 Å, respectively; fig. S7D). Similar results were obtained for the simulations with the nsp5_BA.1_ enzymes that only differ from nsp5_WA1_ by the P132H mutation.

#### GC376 evades resistance from the E166V mutation

The available crystal structures [PDB: 6WTT (*17*) and 8D4M, 8DD9, and 8D4K (*42*)] and simulations of various nsp5:GC376 complexes suggest significantly different interactions at the inhibitor binding sites. GC376 is substantially more flexible than NIR (fig. S8, A to C), primarily due to its P3 benzyl ester group (Fig. 1A). This group can assume highly diverse conformations, with an RMSF for the positional variation of its aromatic ring atoms reaching 2.6 Å (blue arrow, fig. S8B) due to rotation of the P3 benzyl ester group around the bond between the C1 and O2 atoms (fig. S8C). Similarly, but to a lesser extent, the P2 Leu residue of GC376 can assume more conformations than the more constrained Leu analog P2 of NIR with the gem-dimethyl cyclopropane ring (fig. S8, B and C). Therefore, the introduction of the inflexible Val^166^ does not result in any significant repositioning of GC376 as a rigid body because the inherently flexible benzyl ester at the P3 site helps defuse the potential steric conflict by repositioning within the active site [seen during the simulations of WT-nsp5_WA1_:GC376 and E166V-nsp5_WA1_:GC376 (fig. S8, D to F)]. Such flexibility is consistent with our structural data (Fig. 4) and other experimentally determined structures (*17*, *42*). Accordingly, there is no significant change in the interatomic distances of the Cys^145^_Sγ_ in relation to the aldehyde group warhead of GC376 during the simulations of the WT-nsp5_WA1_:GC376 and E166V-nsp5_WA1_:GC376 complexes (fig. S7D). Similar repositioning of the P3 benzyl ester group was observed in the simulations of GC376 with the WT-nsp5_BA.1_ and E166V-nsp5_BA.1_ enzymes.

## DISCUSSION

Antiviral therapies are essential for treating unvaccinated patients or cases of breakthrough SARS-CoV-2 infection. The nsp5 protease in SARS-CoV-2 and other coronaviruses provides a promising drug target due to its essential role in coronavirus replication (*43*). Although Paxlovid (NIR-ritonavir) has shown promising efficacy (*44*), there are reports of viral RNA rebound following Paxlovid treatment (*20*–*24*). Two such instances of rebound in immunocompromised individuals included the E166V mutation, which also appeared during the EPIC-HR clinical trial (*25*, *26*, *30*). These reports, coupled with multiple studies investigating the effects of nsp5 mutations on NIR resistance in cell culture experiments (*27*–*31*), suggest that drug resistance mutations may become a threat to Paxlovid-based therapies, similar to antiviral treatments that target HIV (*45*), hepatitis B virus (*46*), influenza (*47*), and hepatitis C virus (*48*). This necessitates the design of second-generation inhibitors to combat viral strains resistant to NIR.

Regulatory constraints on gain-of-function studies on Paxlovid resistance development through in vitro passaging of infectious SARS-CoV-2 led us to alternative strategies for addressing the development and evasion of NIR resistance. We relied on experience with the structural basis of antiviral resistance (*49*) and on efficient generation of SARS-CoV-2 replicons (>100 total, so far) (*50*). We previously demonstrated that rigid and bulky β-branched amino acid substitutions [such as M184V/I in HIV reverse transcriptase (RT)] sterically hinder the binding of an RT-targeting inhibitor over the substrate, leading to viral resistance against FTC and 3TC (essential drugs against HIV and hepatitis B virus therapies) (*49*). Thus, we hypothesized that amino acid substitutions causing similar steric interactions with NIR would also impart resistance to NIR. The M184V/I substitutions in HIV RT also decrease the enzymatic activity of RT by interfering with substrate binding. Accordingly, we took into consideration the substrate-envelope developed by Shaqra and colleagues to determine the crucial shape of the protease active site and focus on substitutions that avoid clashes with natural substrates while imparting resistance to NIR (*51*). Using available structural data (*33*, *34*), we identified several mutations that could interfere with NIR binding without intruding too far into the substrate-envelope. We then tested these mutations and others identified in parallel passaging experiments using 16 SARS-CoV-2 replicons, each containing one or more mutations (Table 1). Because the Paxlovid EUA was issued on 21 December 2021, amid the fast rise of Omicron, this treatment has been essentially applied by an overwhelming majority on viruses containing the P132H mutation. Thus, we included equivalent constructs of BA.1 and WA1 variants for comparison purposes. Of the substitutions tested, E166V imparted the highest level of resistance (131-fold in WA1 and 66-fold in BA.1 replicons).

### Effects of mutations on BA.1 and WA1 replicon fitness

Early on, it became clear that the E166V mutation not only confers strong NIR resistance but also a significant loss of WA1 replicon fitness (~95%), which can be restored partially by T21I and fully by adding L50F (Fig. 2A). These findings are consistent with a recent independently published work that used recombinant WA1 viruses (*27*–*29*) and with comprehensive mutational analysis of nsp5 (*52*). Although the BA.1 replicons were also resistant to NIR (Table 1), we were surprised to observe only a modest loss in replication fitness (50%) for E166V_BA.1_. Addition of a secondary mutation restored WA1 fitness in this and other studies [L50F (*28*, *29*) or T21I (*29*)] (Fig. 2A). We did not observe major changes in the fitness of the L50F/E166V_BA.1_ or T21I/E166V_BA.1_ replicons compared to E166V_BA.1_ (Fig. 2B), suggesting that these secondary mutations have a beneficial effect either in severely impaired viruses or in the context of WA1 but not Omicron backgrounds. Consistent with the lower fitness of E166V-carrying viruses, this mutation has been reported only 46 times in 15,634,584 sequences submitted to the GISAID database as of January 2025 (~0.00029%). Of those 46 times, 44 were in Omicron strain viruses (i.e., containing P132H), and 13 included the L50F mutation; all of which occurred after the EUA of Paxlovid in December 2021 (Table 2). Recent reports have confirmed the appearance of E166A/V and L50V/E166V in the clinic (*25*, *26*). A recent analysis of SARS-CoV-2 sequences identified E166V as a low-prevalence mutation that is selected for as opposed to appearing de novo during NIR treatment (*53*). If this trend continues, we expect that E166V will increase in prevalence with greater use of Paxlovid while Omicron strains remain the dominant SARS-CoV-2 strains.

### Effect of E166V on activity and dimerization

The decreased fitness of E166V-nsp5 is likely due to the loss of multiple interactions involving Glu^166^ that stabilize the S1 pocket of the active site and are important for the enzymatic activity of nsp5 (*39*, *40*). The Glu^166^ side chain is engaged in a hydrogen bond network with water molecules, Gly^143^, and the N terminus of the opposite protomer (Ser^1^′) to form the critical oxyanion hole (*38*). Glu^166^ directly interacts with the lactam ring of NIR, which mimics the conserved Gln of the consensus sequence. Because dimerization is crucial for nsp5 activity (*38*, *39*, *54*, *55*), disrupting these interactions results in decreased enzymatic function (*54*, *56*). Upon the introduction of the E166V mutation, a loss of interprotomer hydrogen bonds leads to a protein that is primarily monomeric under conditions in which the WT protein readily forms dimers (0% in E166V-nsp5_BA.1_ versus 64% in WT-nsp5_BA.1_) (Fig. 6). Crystal structures of both E166V-nsp5_BA.1_ and E166V-nsp5_WA1_ show that, in the absence of interactions with the Glu^166^ side chain, the Ser^1^′ residue changes orientation (fig. S5). These data agree with both recent MD studies suggesting that Val^166^ would destabilize nsp5 dimerization through disruption of dimer interactions S1′-F140 and R4′-E290 (*28*) and native mass spectrometry experiments with E166V-nsp5 (*57*).

### Differences in Omicron BA.1 and WA1 fitness

Our data suggest that the difference in replication capacity between BA.1 and WA1 E166V is due to the stability of nsp5 dimers. P132H does appear to have a stabilizing effect on the nsp5 dimer (fig. S9) as a larger proportion of the WT-nsp5_BA.1_ remained dimeric at a lower concentration compared to WT-nsp5_WA1_ [72% compared to 56%, respectively, at 15 μM (table S3)]. In addition, the calculated averaged molecular weight for E166V-nsp5_BA.1_ was higher than E166V-nsp5_WA1_ [43 kDa versus 40 kDa (Fig. 6 and table S3)]. To elucidate why E166V has dissimilar deleterious effects when nsp5 has a Pro^132^ (WA1) or His^132^ (BA.1), we introduced only the P132H nsp5 mutation into WA1 replicons, instead of all the 13 Omicron-related nsp mutations (Fig. 2). This was conducted in E166V and L50F/E166A/L167F replicons. Introducing P132H into E166V_WA1_ or H132P into E166V_BA.1_ did not significantly change replicon fitness either way indicating that differences at residue 132 alone do not account for the difference in fitness between E166V_WA1_ and E166V_BA.1_. Notably, we show that, for L50F/E166A/L167F, the observed fitness differences between L50F/E166A/L167F_WA1_ and L50F/E166A/L167F_BA.1_ can be reversed with the single mutation at residue 132 (Fig. 2). In both cases, changing residue 132 did not significantly affect resistance to NIR (Table 1).

### NIR resistance and how to overcome it

Virological data in Table 1 demonstrated that the E166V mutation imparts strong resistance to NIR in both BA.1 (~65-fold) and WA1 (~130-fold) replicons. Testing additional replicons (16 in total) showed that resistance was even stronger in L50F/E166V and T21I/E166V. IC_50_ and nanoDSF experiments confirmed this increased resistance for both WA1 and BA.1 E166V-nsp5 purified proteins in vitro (tables S1 and S2). Our data demonstrate that NIR resistance arises not because the inhibitor cannot enter the active site, but rather because it cannot efficiently form a stable covalent bond. In BLI experiments, association with NIR occurs at roughly the same rate (*k*_on_) in both WT-nsp5_BA.1_ and E166V-nsp5_BA.1_, but E166V-nsp5_BA.1_ has a significantly lower *K*_d_ due to a faster *k*_off_ compared to WT-nsp5. NIR does not appear to dissociate from WT-nsp5_BA.1_ over the course of the experiment, consistent with covalent bond formation (Fig. 5A and Table 3). Crystallization of E166V-nsp5_BA.1_ in complex with NIR shows the inhibitor in the active site in two binding modes with the majority positioned further away from the catalytic sulfur (Cys^145^_Sγ_) (~70%), where it cannot form a covalent bond (Fig. 3B). BLI data support noncovalent binding of NIR with E166V-nsp5_BA.1_ (Fig. 5C and Table 3). MD analysis indicated that NIR binds the active site as a relatively rigid body during the simulations (fig. S7, D and E). Although this notable rigidity of NIR imparts strong binding to WT-nsp5 and is consistent with the low nanomolar antiviral EC_50_ values (Table 1), it comes at the expense of conformational flexibility. Simulation of the E166V-nsp5_BA.1_:NIR complex suggests sterically driven repositioning of the bulky NIR *tert-*butyl (P3 group), which protrudes outside the substrate envelope (*58*), by the also rigid and bulky β-branched Val^166^ (fig. S7D). Although relocation of the P3 *tert*-butyl alleviates the steric conflict with Val^166^, the overall rigidity of NIR causes repositioning of the lactam ring (P1 group) at the catalytic site and concomitant reorientation of the NIR P1′ cyano group warhead. These changes appear to also affect the position of catalytic residue His^41^. Thus, the rigid body movement of NIR, which is the result of its limited conformational flexibility, results in suboptimal positioning of the warhead in relation to Cys^145^. A computational work with this complex has also revealed that changing how the inhibitor sits in the active site increases the free energy cost of the covalent bond formation reaction, thus decreasing the efficiency of NIR covalent binding at the E166V-nsp5_BA.1_ active site (*59*). This negative impact may be further augmented by the loss of the hydrogen bond interactions of Glu^166^, which deforms part of the active site and changes the electrostatics surface of the active site, both of which likely affect the affinity of NIR binding as recently suggested (*28*, *53*, *59*). On the basis of this model, we predict that other β-branched mutants (E166T and E166I) would also have similar effects on NIR resistance.

### GC376 evades E166V-based resistance through strategic torsional flexibility and structural adaptation or “wiggling and jiggling”

Virological data in Table 1 show that, unlike the strong NIR resistance (>50-fold to >100-fold) conferred by E166V, L50F/E166V, or T21I/E166V in both BA.1 and WA1 replicons, there is a notable lack of resistance of the same replicons against GC376 (0.6-fold to 2-fold) or recombinant enzymes in vitro (table S1). Binding studies show similar *K*_d_ values for WT-nsp5 and E166V-nsp5 with similar *k*_off_ values identical to that of WT-nsp5 with NIR (Fig. 5, B and D, and Table 3). When crystallized in complex with E166V-nsp5_BA.1_, GC376 forms a covalent bond with Cys^145^; to relieve potential steric interactions with the β-branched, bulky, and inflexible Val^166^, its benzyl group assumes multiple conformations through strategic repositioning from its location in the WT-nsp5_BA.1_ active site (Fig. 4). Similar repositioning of the benzyl group has been observed in the crystal structures of other nsp5:GC376 complexes containing various mutations (*17*, *60*) (PDB ID: 6WTT versus 8D4M, 8DD9, and 8D4K). Hence, the strategic torsional flexibility of GC376 allows it to avoid steric conflict and structurally adapt in a changing pocket, enabling covalent binding, inhibition of E166V-nsp5, and evasion of resistance.

The role of inhibitor flexibility in nsp5 drug resistance was confirmed by MD simulations (figs. S7 and S8) that show sharp differences in the flexibility of NIR versus GC376. GC376 showed increased torsional and conformation flexibility at the P4 benzyl ester group of GC376. This group moves significantly during the simulation adopting diverse conformations to avoid Val^166^ (fig. S8F). Thus, strategic design of antivirals with flexible, adaptable structures could be a helpful approach to minimize steric hindrance-based drug resistance. A similar strategy, known as “wiggling and jiggling,” had been proposed by Das and colleagues in the design of second-generation non-nucleoside RT inhibitors (NNRTIs) such as rilpivirine that can bind in multiple conformations at the evolving NNRTI-binding pocket of HIV RT and avoid drug resistance mutations (*60*).

Compliance to gain-of-function restrictions prevented us from conducting passages using infectious virus to identify resistance mutations. However, we overcame this limitation through constructing 16 SARS-CoV-2 replicons to assess the effect of drug resistance mutations on SARS-CoV-2 replication (*50*). Although the replicon system does not recapitulate the SARS-CoV-2 replication cycle in its entirety, it has been used extensively for assessing the effect of antivirals and mutations on replication capacity (*61*, *62*). We were also unable to passage viruses containing the E166V mutation to investigate possible compensatory mutations that could arise naturally to mitigate the fitness loss associated with this mutation. We did include mutations identified by passaging studies associated with NIR resistance and accompanying compensatory mutations (*27*–*31*, *42*). The flexibility of the replicon system will allow us to easily investigate clinical mutations that emerge during treatment as they are the most reliable source of relevant drug resistance information.

In conclusion, we independently identified and characterized the key mutation (E166V) that confers strong NIR resistance in both Omicron and WA1 strains. We showed fitness differences across replicons from the different strains, which may lead to a variability in barriers to resistance between Omicron and non-Omicron strains. This is because of differences in dimerization efficiency. We propose increasing conformational flexibility to allow for “wiggling and jiggling” in the active site to avoid steric clashes as a strategy for designing second-generation antivirals against NIR-resistant viral strains.

## MATERIALS AND METHODS

### Cells 

Human embryonic kidney (HEK) 293T/17 cells (CRL-11268, American Type Culture Collection, Manassas, VA, USA) were cultured in Dulbecco’s modified Eagle’s medium ( #10313-021, Gibco, Waltham, MA, USA) with 10% Serum Plus II (Sigma-Aldrich, St. Louis, MO, USA) supplemented with penicillin (100 U/ml), streptomycin (100 μg/ml; #400-109, Gemini Bioproducts, West Sacramento, CA, USA), and 2 μM l-glutamine (#25030-081, Gibco), in a humidified incubator at 37°C with 5% carbon dioxide.

### Plasmids

A previously described cell-based luciferase complementation reporter assay was used to assess SARS-CoV-2 nsp5 mutations and susceptibility to inhibitors (*63*). We used versions of the nsp5-S-L-GFP reporter plasmid that were either WT (of Washington or WA1 strain), catalytically inactive (C145A), or carrying mutations at other nsp5 positions (F140I, M165D, E166L, N142L, E166V, and E166I in the WA1 background). Mutants were generated by site-directed mutagenesis using QuikChange II (Agilent, Santa Clara, CA, USA) and validated via Sanger sequencing (GeneWiz, Chelmsford, MA, USA).

SARS-CoV-2 replicons (SARS-2R_mNG_NeoR_NL) of WA1 and Omicron BA.1 were constructed either in the WT background (WT_WA1_ and WT_BA.1_) or in the presence of putative drug resistance mutations at the 21, 50, 132, 142, 166, and 167 sites of nsp5 as previously described (*50*). Construction of the N expression vectors was as previously described (*50*). All construct sequences were confirmed by Sanger sequencing (Azenta, Chelmsford, MA, USA) or full-length sequencing (Plasmidsaurus, Eugene, OR, USA). Sequencing results were analyzed with Lasergene/DNASTAR software (Madison, WI, USA).

For biochemical assays, SARS-CoV-2 nsp5 was cloned into the pGEX-6P-1 vector using BamHI and XhoI and then synthesized commercially (GenScript, Piscataway, NJ, USA). This construct contains an N-terminal GST-tag and a C-terminal His10-tag. A native N terminus is attained during expression through an autoprocessing site corresponding to the cleavage between nsp4 and nsp5 in the viral polyprotein (SAVLQ ↓ SGFRK, where ↓ denotes the cleavage site). The C-terminal His10-tag is preceded by a human rhinovirus 3C (HRV-3C) protease cleavage sequence (VTFQ↓GP) for cleavage. The E166V mutation was introduced using QuikChange site-directed mutagenesis (Agilent, Santa Clara, CA, USA). Sequences were validated via Sanger sequencing (Azenta, Chelmsford, MA, USA).

### Cell-based luciferase complementation reporter assay method

The nsp5-S-L-GFP reporter system contains the nsp5 sequence followed by a porcine teschovirus 2A cleavage signal and a NanoLuc luciferase sequence separated by the nsp4/nsp5 cut site (*63*). Green fluorescent protein (GFP) is also included to act as a transfection control. Functional nsp5 cleaves the nsp4/nsp5 site, rendering NanoLuc inactive. By contrast, inactive mutants do not cleave the nsp4/nsp5 site, resulting in measurable NanoLuc activity. HEK293T/17 cells were seeded onto a 6-well plate and then transfected 24 hours later using X-tremeGENE HP (Roche, Basel, Switzerland). After transfection (24 hours) cells were re-seeded into a 96-well plate (40,000 cells per well) containing serial dilutions of inhibitors. After 24 hours, transfection efficiency was determined by counting GFP-positive cells. Cells were then lysed, and NanoLuc activity was measured using the NanoGlo Luciferase Assay System (Promega, Madison, WI, USA). Luciferase activity was normalized based on the number of GFP-positive cells in each well and to the NanoLuc level of the catalytically inactive C145A mutant (*64*). Significance was assigned using a one-way analysis of variance (ANOVA) with Tukey statistical test.

### Replicon fitness and dose-response

SARS-CoV-2 replicons (SARS-2R_mNG_NeoR_NL) of WA1 and Omicron BA.1 strains were constructed in the absence of mutations (WT_WA1_ and WT_BA.1_) or in the presence of mutations at the 21, 50, 132, 142, 166, and 167 sites of nsp5 as we have previously described (*50*). HEK293T/17 cells seeded in a 6-well plate were transfected with 1 μg of replicon plasmid (SARS-2R) using jetPRIME transfection reagent (Polyplus transfection, Illkirch-Graffenstaden, France). At 16 hours posttransfection, cells were trypsinized and then seeded into 96-well plates and treated with serial dilutions of antivirals. NanoLuc luciferase assays were performed 48 hours posttreatment of SARS-2R with individual antivirals. EC_50_ values were determined using a nonlinear regression curve fit with variable slope in GraphPad Prism 9.2.0 (GraphPad, San Diego, CA, USA). Replicon fitness was determined by comparison of reporter gene expression of DMSO-treated mutants to WT replicons when equal amounts of transfected nucleic acid were used (based on Nanodrop measurements). A two-way ANOVA was used to analyze the replicon fitness assay. Errors provided for values in tables are SDs based on three to four biological replicates.

### Expression and purification of nsp5 for biochemical assays

E166V_WA1_ was generated from the WT plasmid using QuikChange II site-directed mutagenesis. WT_WA1_ and E166V_WA1_ proteins were expressed in *Escherichia coli* BL21 (DE3) by growing 100 ml of starter cultures [containing chloramphenicol (34 mg/ml) and carbenicillin (100 mg/ml)] and allowed to grow overnight. Twenty-five milliliters of the starter culture was added to 1 liter of LB, containing equal amounts of aforementioned antibiotics, and grown to an optical density (OD) of ~0.8, after which protein expression was induced with 0.2 mM isopropyl-β-d-thiogalactopyranoside. Bacteria were incubated at 18°C overnight with shaking. Bacteria were then pelleted by centrifuging for 30 min at 3000 rpm, and cell pellets were stored at −20°C.

Purification was performed using TALON resin. Bacteria pellets were resuspended in 10 ml of lysis buffer [25 mM Tris (pH 8.0), 300 mM NaCl, 5 mM β-mercaptoethanol (β-Me), and 4 mM MgCl_2_ with lysozyme (0.15 mg/ml)] for 30 min and then lysed using sonication. Cell debris was then pelleted by centrifugation (14,000 rpm at 4°C for 30 min), and the supernatant was then treated with 0.05% polyethylenimine (PEI) and centrifuged again. An equal volume of saturated ammonium sulfate was added to the supernatant and incubated at 4°C overnight. Protein was pelleted by centrifugation (14,000 rpm at 4°C for 30 min) and then resuspended in lysis buffer and centrifuged again before the addition of 1 to 2 ml of preequilibrated TALON resin (Cytiva, Marlborough, MA, USA). Supernatant and TALON resin were incubated at 4°C for 2 hours before loading onto a gravity flow column. The resin was washed using lysis buffer with 0, 20, 50, and 100 mM imidazole (pH 8.0) and eluted using lysis buffer with 300 mM imidazole (pH 8.0). For His-tagged proteins for BLI, the protein was dialyzed and stored in 25 mM Tris (pH 8.0), 100 mM NaCl, 5 mM β-Me, and 4 mM MgCl_2_. Otherwise, the C-terminal His10-tag was cleaved during dialysis using a His10-tagged human rhinovirus 3C (HRV-3C-His) protease. After dialysis, the protein was incubated with TALON resin a second time to remove the HRV-3C-His and cleaved His10-tag, and the flow-through was collected and stored in 25 mM Tris (pH 8.0), 100 mM NaCl, 5 mM β-Me, and 4 mM MgCl_2_.

### In vitro IC_50_ assay

Nsp5 activity was determined by measuring changes in fluorescence on peptide substrates carrying both a fluorophore, MCA (4-methylcoumaryl-7-amide), and a quencher, DNP (2,4-dinitrophenyl), as previously described (*65*). Measurements were performed in 20 mM Bis-Tris (pH 7.0) in a well volume of 100 μl of a peptide substrate that includes the nsp5 cleavage site between nsp4 and nsp5 proteins (nsp4-5) (-AVLQ ↓ SGFR[K(DNP)]K-NH_2_ (MilliporeSigma, Milwaukee, WI, USA; >95%). Assays were performed by incubating 2 μM enzyme with a serial dilution of the inhibitor for 10 min and adding 500 μM substrate immediately before reading. Activity was measured for 30 min on a Cytation 3 plate reader using a monochromator (Ex: λ = 322 nm/Em: λ = 381 nm). To determine the IC_50_, the slope of the linear region (the first 5 min) was determined and normalized to the slope of the uninhibited enzyme. These values were then used to create a dose-response curve and calculate IC_50_ values using GraphPad Prism 9.2.0 (GraphPad, San Diego, CA, USA). Errors provided for values in tables are SDs based on three biological replicates.

### Sequence analysis

Sequences were taken from the EpiCoV database, the most comprehensive database of SARS-CoV-2 sequences currently available curated by GISAID (*66*). The curated “allprot” protein sequence alignment, obtained on December 2021 and January 2025, was analyzed using in-house Python scripts from GISAID (Table 2). Nsp5 sequences for each protein were extracted from this dataset and filtered to remove sequences containing ambiguous residues (those for which one or more nucleotides were unassigned) or insertions and deletions. The filtered sequences (7,313,022 total sequences December 2021 and 15,634,584 total sequences January 2025) were then screened for specific amino acid changes related to this work.

### nanoDSF measurements

The thermal stability of the nsp5 proteins with NIR or GC376 was measured using label-free differential scanning fluorimetry (nanoDSF). Samples of 15 μM protein were incubated with 20 μM DMSO, NIR, or GC376 for 10 min before measurement using a NanoTemper Tycho instrument (NanoTemper Technologies GmbH, München, Germany). Monitoring the intrinsic fluorescence of aromatic residues at 330 and 350 nm measures protein stability and unfolding. Inflection temperatures (*T*_i_) were determined based on triplicate measurements for each protein-drug combination.

### Crystallization

For the NIR complex, apo E166V-nsp5_BA.1_ crystals grew at 18°C in hanging drops containing a protein (~2 mg/ml), 14 to 18% polyethylene glycol, molecular weight 3350, and 0.1 to 0.3 M ammonium formate. Clusters of crystal plates appeared after 24 hours and continued growing for ~48 hours. Before freezing, apo E166V-nsp5_BA.1_ crystals were soaked in a solution containing 10 mM NIR, 5% DMSO, 10% glycerol, and well solution for 3 hours at 30°C. After soaking, crystals were cryoprotected in a solution containing 20% glycerol before flash freezing in liquid nitrogen.

For the GC376 complexes, E166V-nsp5_BA.1_ or E166V-nsp5_WA1_ (2 mg/ml) was cocrystallized with 2 mM GC376 and 5% DMSO using the same buffer conditions as apo crystals. Crystals were soaked in 2.5 mM GC376, 5% DMSO, and 10% glycerol for 20 min at room temperature before freezing in a solution of 20% glycerol.

### Data collection and refinement

X-ray diffraction data of the E166V-nsp5_BA.1_:NIR and E166V-nsp5_BA.1_:GC376 complexes were collected at SERCAT beamline 22-ID at the Advanced Photon Source. Diffraction data of the E166V-nsp5_WA1_:GC376 complex was collected at the National Synchrotron Light Source II (NSLS-II) on beamline 17-ID-2 (FMX) (*67*). Data were processed by XDS (*68*) and scaled using Aimless (*69*). The space group of all structures was determined to be *I*2 (or *C*2), with one monomer in the asymmetric unit. Molecular replacement was performed using Phaser (*70*) with PDB: 9EEI (for E166V-nsp5_BA.1_:NIR and E166V-nsp5_WA1_:GC376) and PDB: 7TOB (for E166V-nsp5_BA.1_:GC376) as an initial model. Several rounds of iterative model building and refinement were carried out using Coot (*71*) and Phenix (*72*), respectively. Structure validation of final models was performed using Molprobity (*73*) and the PDB validation server. Statistics for the three structures published here are included in table S4. The figures showing structural information were generated in UCSF ChimeraX (*74*).

### BLI binding studies

Frozen aliquots of nsp5-His proteins were diluted to 3 μM in BLI buffer [25 mM Tris (pH 7.5) with 100 mM NaCl, 5 mM β-Me, and 4 mM MgCl_2_, 5% glycerol, and 0.5% Tween 20]. NTA (Ni-NTA) Dip and Read Biosensors (FortéBio, #18-5101) were first hydrated in 200 μl of buffer for 10 to 30 min before sample loading. All experiments were performed in 96-well microplates (Greiner, 655209), agitated at 1000 rpm, at 25°C, and a volume of 200 μl per well.

The BLI protocol was modified from the default parameters in the Octet BLI Discovery program (version 13.0.0.17, Sartorius). Experiments were initiated with a 120-s baseline step, followed by loading of nsp5 for 400 s. The nsp5-loaded probe was washed twice in BLI buffer for 60 s. Probes were then dipped into a 200-μl solution of BLI buffer containing 100, 75, or 50 μM NIR or GC376 for 50 s of association time and then 100 s of dissociation time in a well containing only BLI buffer.

Octet Analysis Studio (version 13.0.0.32, Sartorius) was used to perform double background subtraction by subtracting the signal from a protein-only control (i.e., DMSO without inhibitor) and a parallel protein-free reference biosensor to account for nonspecific binding. Response curves were aligned to the beginning of the association step using baseline interstep correction. Association and dissociation curves were fit with a continuous 1:1 protein:ligand binding model. The resulting *K*_d_, *k*_on_, and *k*_off_ values were determined using global fitting of three independent replicates for each inhibitor concentration. In the case of covalent inhibitors, *k*_off_ reaches the limit of detection, so upper limits for *K*_d_ and *k*_off_ are provided in Table 3. Residual plots are shown in fig. S10.

### Size exclusion chromatography coupled to multiangle light scattering

SEC-MALS experiments were performed using either 60 or 15 μM WT-nsp5_BA.1_ or E166V-nsp5_BA.1_ in storage buffer [20 mM Tris (pH 7.5), 100 mM NaCl, 4 mM MgCl_2_, and 5 mM β-Me] with 0.02% sodium azide. Experiments were also run using 15 μM protein with a 10-fold excess of NIR or GC376. All SEC-MALS experiments were performed by injecting a 100-μl sample at a flow rate of 0.25 ml/min at room temperature using an analytical Superdex 200 Increase 10/300 column (GE Healthcare) with in-line ultraviolet (UV)–visible (Waters Corporation, USA) and MALS (Wyatt Technology Inc., Santa Barbara, CA) detectors. Data were analyzed using the ASTRA V.7.1.2 program (Wyatt Technologies) to determine the mean and SD of the molecular mass of peaks resolved by SEC. In the case of multiple peaks of interest, the software can also be used to estimate the relative population of each peak in the chromatogram.

### MD simulations

Initial structural coordinates for nsp5 in complex with GC376 (PDB ID: 7TGR) and NIR (PDB ID: 7RFW) were retrieved from the PDB (*13*, *36*). These structures were prepared for MD simulations using the Maestro modeling environment within the Schrödinger Software suite (Schrödinger Release 2022-2: Schrödinger, LLC, New York, NY, 2021). Briefly, the protein preparation workflow was used to add hydrogens, assign disulfide bonds, remove cocrystallizing small molecules and ions, and fill in missing side chains (*75*). Hydrogen bond (H-bond) assignments were optimized to resolve overlap; protonation states were assigned using PROPKA (*76*). For the respective ligands, protonation and charge states were calculated at pH 7.4 ± 2.0 and the initial state of the ligand was selected based on calculating the number of hydrogen bonds and the Epik penalty score (Schrödinger Release 2022-2: Schrödinger, LLC, New York, NY, 2021) (*77*, *78*). Last, a restrained minimization was performed using the OPLS4 force field (*79*). The prepared nsp5-inhibitor complexes were then solvated in a 12 Å–by–12 Å–by–12 Å box using the TIP3P water model (*80*). Counterions were added to neutralize the charge of the system, and additional Na^+^ and Cl^−^ ions were added to a final concentration of 150 mM. MD simulations were performed using the Desmond MD simulation package within the Schrödinger Software suite (Schrödinger Release 2022-2: Desmond Molecular Dynamics System, D. E. Shaw Research, New York, NY, 2021. Maestro-Desmond Interoperability Tools, Schrödinger, New York, NY, 2021). The model systems were initially relaxed using Maestro’s default relax model system protocol and equilibrated with a 5-ns simulation run under isothermal-isobaric (NPT) ensemble conditions (temperature: 310 K; pressure: 1.01325 bar). The coordinates of these model systems were then used as the starting point for 100-ns runs. All simulations were performed with a 2-fs time step, and coordinates were recorded at an interval of 20 ps. Simulation event analysis and simulation interaction diagram tools within Maestro were used for trajectory analysis.
