# Supplementary material for: Strategy to overcome a nirmatrelvir resistance mechanism in the SARS-CoV-2 nsp5 protease
Source: Sci Adv. 2025 Jun 6;11(23):eadv8875. doi: 10.1126/sciadv.adv8875 (PMC12143351; doi:10.1126/sciadv.adv8875)
Supplement: Supplementary file 1 — Figs. S1 to S10 Tables S1 to S4 [file sciadv.adv8875_sm.pdf]

Science Advances
AAAS

Supplementary Materials for

Strategy to overcome a nirmatrelvir resistance mechanism in the SARS-CoV-2 nsp5 protease

Grace Neilsen *et al.*

Corresponding author: Stefan G. Sarafianos, stefanos.sarafianos@emory.edu; Shuiyun Lan, shuiyun.lan@emory.edu

Sci. Adv. **11**, eadv8875 (2025)
DOI: 10.1126/sciadv.adv8875

This PDF file includes:

Figs. S1 to S10
Tables S1 to S4

Supplementary Figures

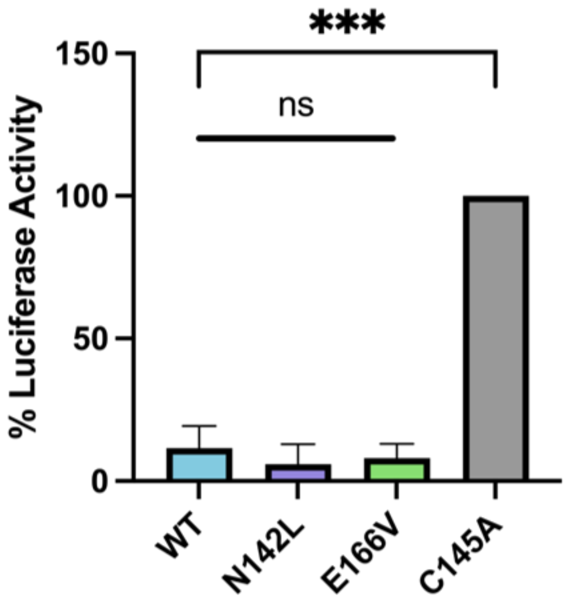

Fig. S1. Fitness of active site mutants measured in the absence of inhibitor. NanoLuc activity was normalized to the number of GFP (+) cells to control for transfection efficiency and the signal from the C145A-nsp5$_{WA1}$. Both N142L and E166V showed lower NanoLuc activity (i.e., higher enzymatic activity) than the C145A nsp5 and comparable activity to the WT-nsp5$_{WA1}$ (One-way ANOVA with Tukey $p = 0.037$ (n=3)). Error bars indicate standard deviation.

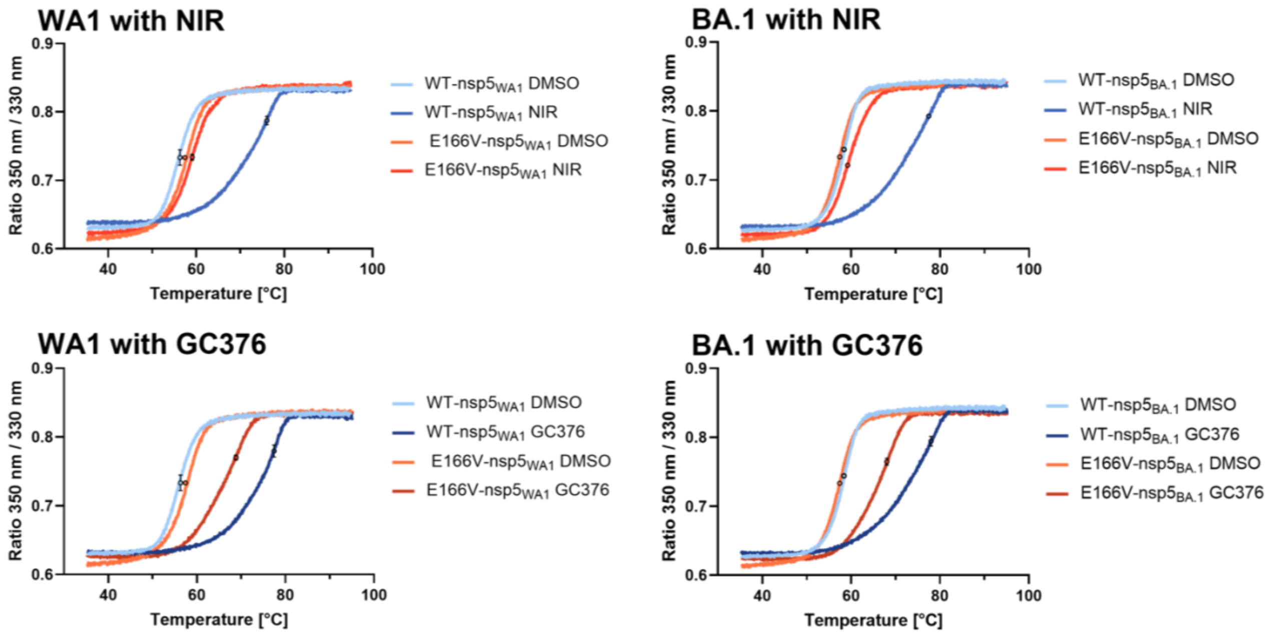

Fig. S2. Thermal Stability of nsp5 proteins in the presence of inhibitors. Average nanoDSF thermal stability curves and inflection temperature (T_i) of $nsp5_{WA1}$ and $nsp5_{BA.1}$ proteins with DMSO, NIR, or GC376. Error bars indicate standard deviation based on n = 3 replicates.

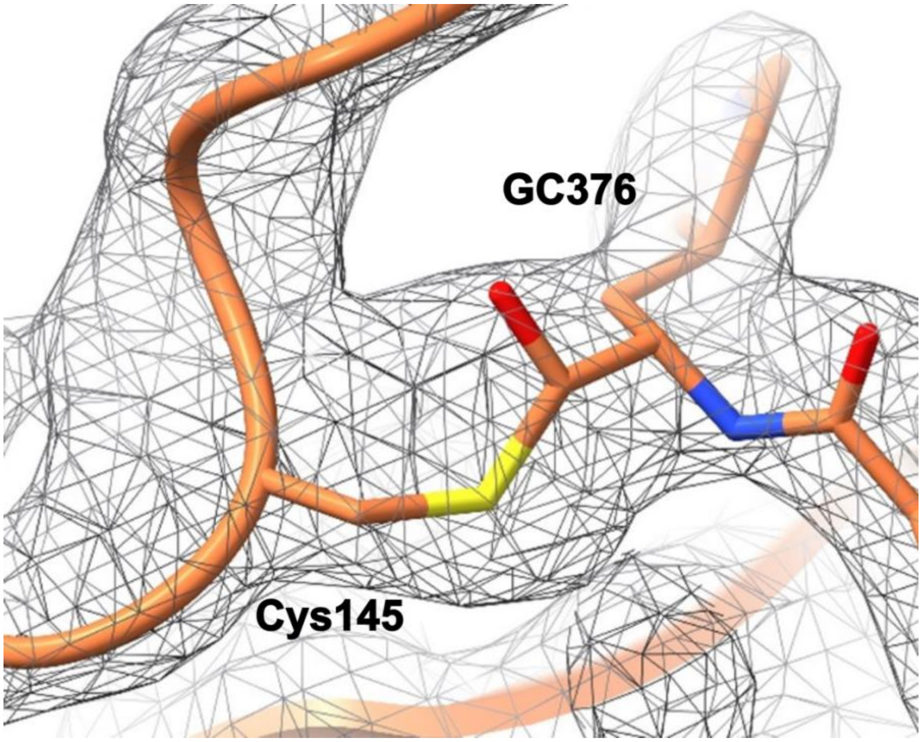

Fig. S3. Covalent bond formation between E166V-nsp5$_{BA.1}$ and GC376. Electron density extends from Cys145 to the warhead of GC376 in E166V-nsp5$_{BA.1}$. A 2Fo-Fc map of E166V-nsp5$_{BA.1}$ shown as grey mesh (contoured at $\sigma = 1.0$).

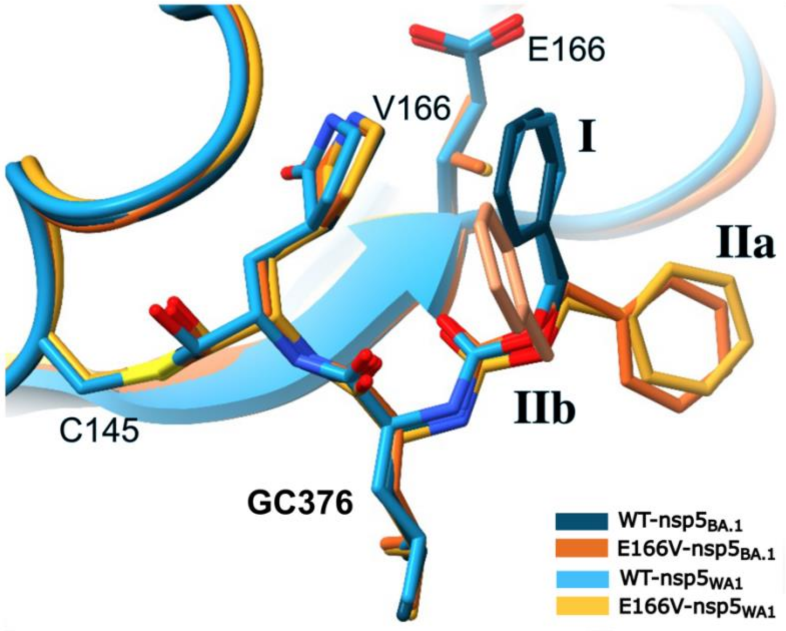

Fig. S4. Overlay of WT- and E166V-nsp5 proteins in complex with GC376. The benzyl groups of WT-nsp5$_{BA.1}$ (dark blue, PDB ID: 7TOB) and WT-nsp5$_{WA1}$ (light blue, PDB ID: 7JSU) both occupy position **I**, and the benzyl groups of E166V-nsp5$_{BA.1}$ (orange) and E166V-nsp5$_{WA1}$ (yellow) occupy the same position in **IIa**. In the E166V-nsp5$_{BA.1}$ structure, a secondary conformation of the benzyl ring is also seen in **IIb** (tan).

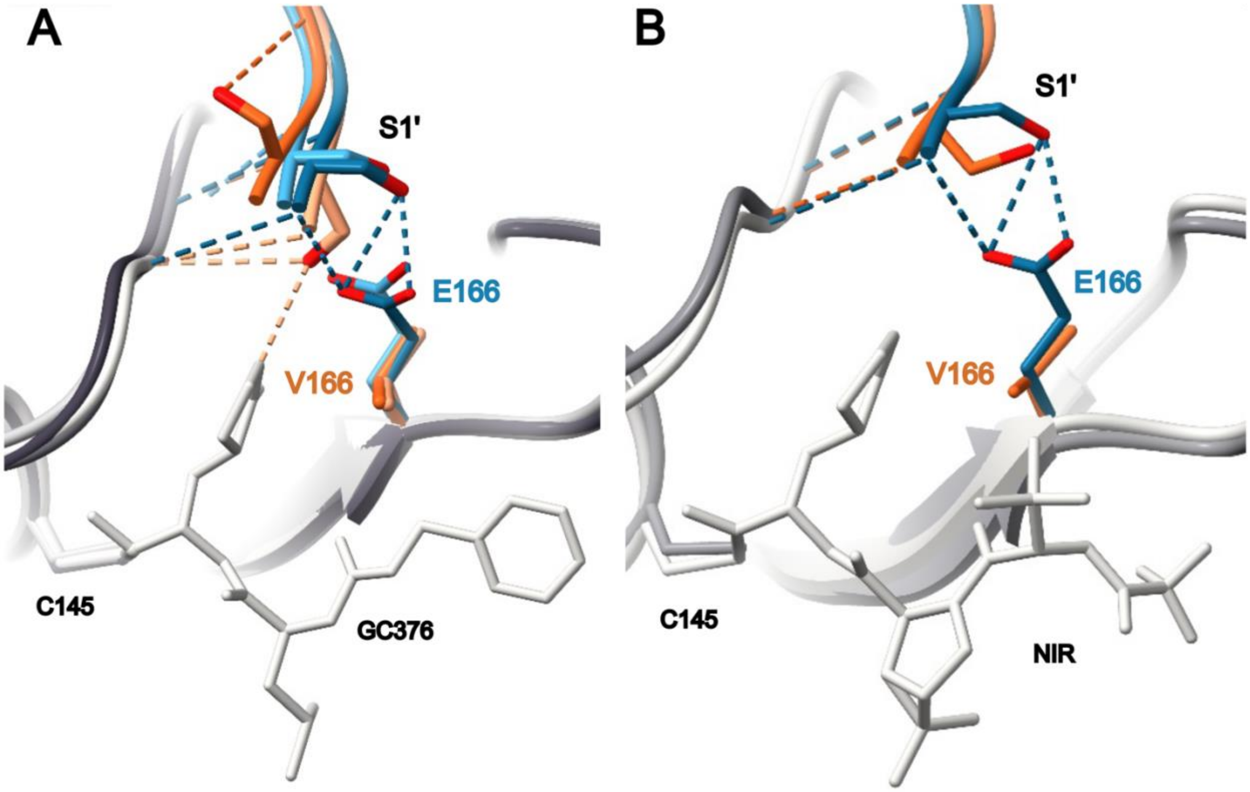

Fig. S5. Changes in the S1' position of the neighboring nsp5 protomer. (A) Alignment of the WT- and E166V-nsp5$_{WA1}$ and WT- and E166V-nsp5$_{BA.1}$ in complex with GC376, including the N-terminus of the opposite protomer (S1'). Residue 166 and opposite protomer shown in orange for E166V-nsp5$_{BA.1}$:GC376; tan for E166V-nsp5$_{WA1}$:GC376; dark blue for WT-nsp5$_{BA.1}$:GC376 (PDB ID: 7TOB); light blue for WT-nsp5$_{BA.1}$:GC376 (PDB ID: 7SNA). Dashed lines indicate hydrogen bonds formed by S1' of the respective proteins. The GC376 inhibitor of E166V-nsp5$_{WA1}$:GC376 is included as a reference, and all others have been removed for clarity. **(B)** Alignment of WT- and E166V-nsp5$_{BA.1}$ in complex with NIR (residue 166 and opposite protomer shown in orange for E166V-nsp5$_{BA.1}$:NIR; dark blue for WT-nsp5$_{BA.1}$:NIR (PDB ID: 7TLL)). Dashed lines indicate hydrogen bonds formed by S1' of the respective proteins. The NIR inhibitor of WT-nsp5$_{BA.1}$:NIR is included as a reference.

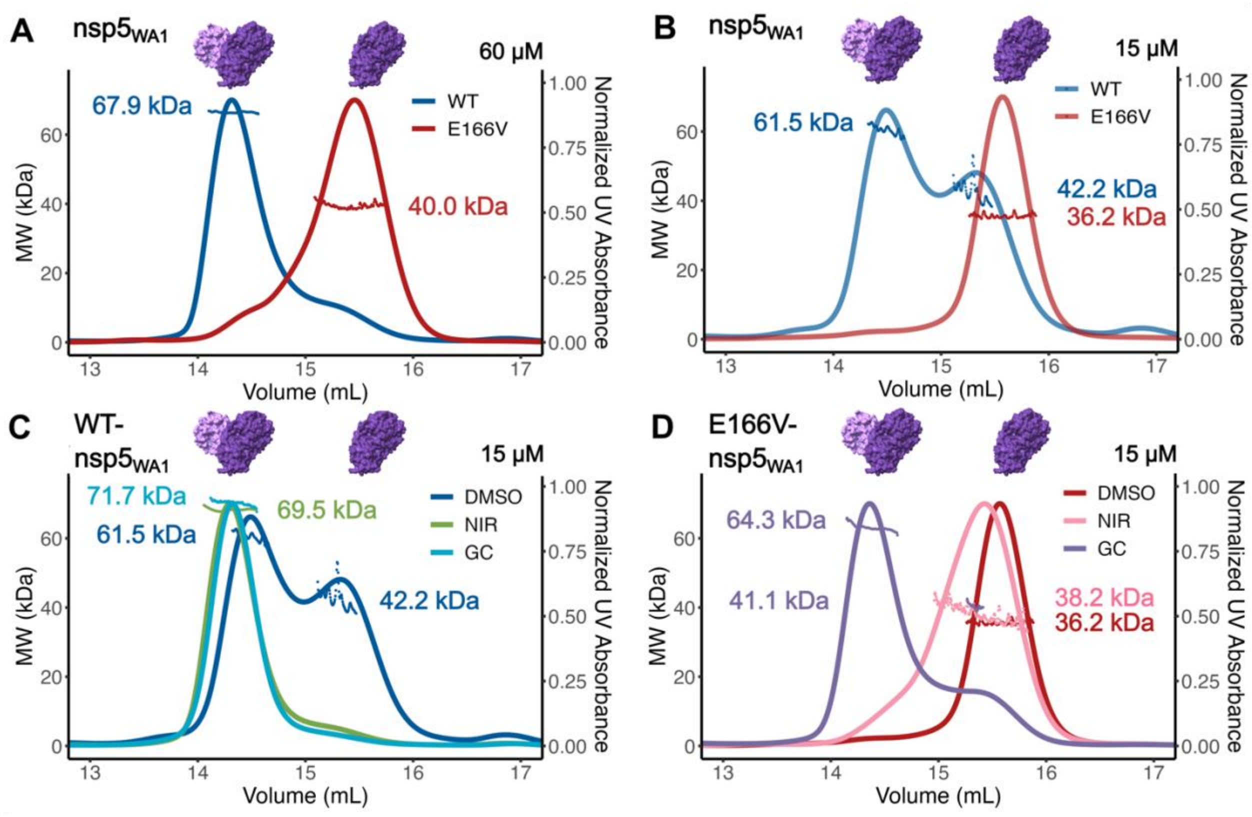

Fig. S6. Effect of E166V and inhibitors on nsp5$_{WA1}$ dimerization. Size-exclusion chromatography coupled to multiangle light scattering (SEC-MALS) with WT-nsp5$_{WA1}$ and E166V-nsp5$_{WA1}$ proteins at 60 μM (**A**) and 15 μM (**B**). **C)** WT-nsp5$_{WA1}$ (15 μM) in the presence of DMSO, NIR, or GC376. **D)** E166V-nsp5$_{WA1}$ (15 μM) in the presence of DMSO, NIR, or GC376. Points indicate the molecular weight (MW) calculated based on the light scattering and UV absorbance (normalized UV absorbance shown as a trace). The expected MWs are ~33.8 kDa for a pure monomeric population and ~67.6 kDa for a pure dimeric population.

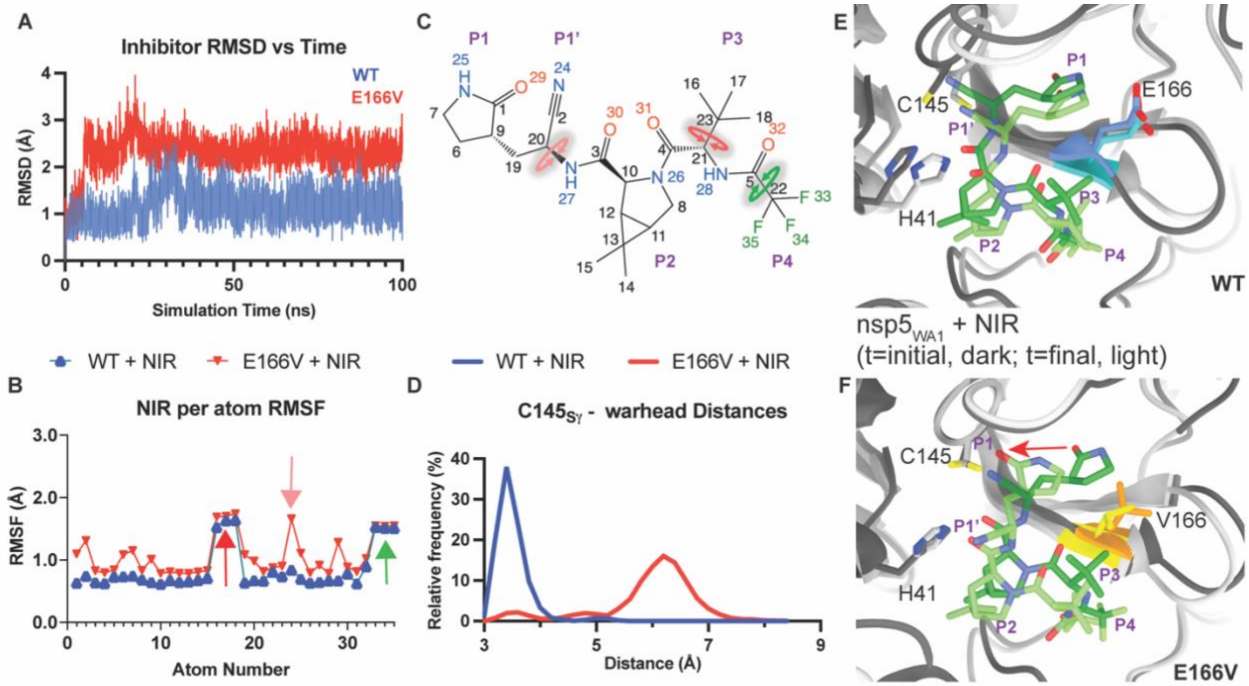

Fig. S7. **Conformational rigidity results in changes in NIR binding within the WT-nsp5$_{WA1}$ and E166V-nsp5$_{WA1}$ active sites. (A)** Average RMSD of NIR binding in the WT- and E166V-nsp5$_{WA1}$:NIR complexes, computed by aligning at every time point to the reference nsp5$_{WA1}$ backbone structure at the beginning of the simulation, then computing the RMSD of non-hydrogen NIR atoms. The WT- and E166V-nsp5$_{WA1}$:NIR simulations are in blue and red. **(B)** Positional RMSF of NIR atoms, numbered as in (C). Red and green arrows indicate the freely rotating P3 and P4 groups shown in (C). These values represent the internal atomic fluctuations of NIR at the end of the simulation compared to their starting positions. **(C)** 2D representation of NIR. Atom numbers correspond to the positions plotted on the horizontal axis in (B). Inhibitor sites are shown in purple, as previously defined. Rotating bonds corresponding to the colored arrows in (B) are indicated by circular arrows of the same color. **(D)** Frequency distribution (in percentage of total simulation time) of interatomic distances of the catalytic C145$_{S\gamma}$ in relation to the reactive cyano carbon [C2 in (C)]. **(E, F)** Superimposition of WT-nsp5$_{WA1}$:NIR (E) and E166V-nsp5$_{WA1}$:NIR (F) complexes at the beginning and end of the simulation (dark *vs.* light colors, respectively; E166 is in blue, V166 in yellow, rest of nsp5 in gray, NIR in green). Catalytic residues H41 and C145 are labeled. The red arrow in (F) indicates the lactam ring repositioning at the end of the simulation compared to its starting position.

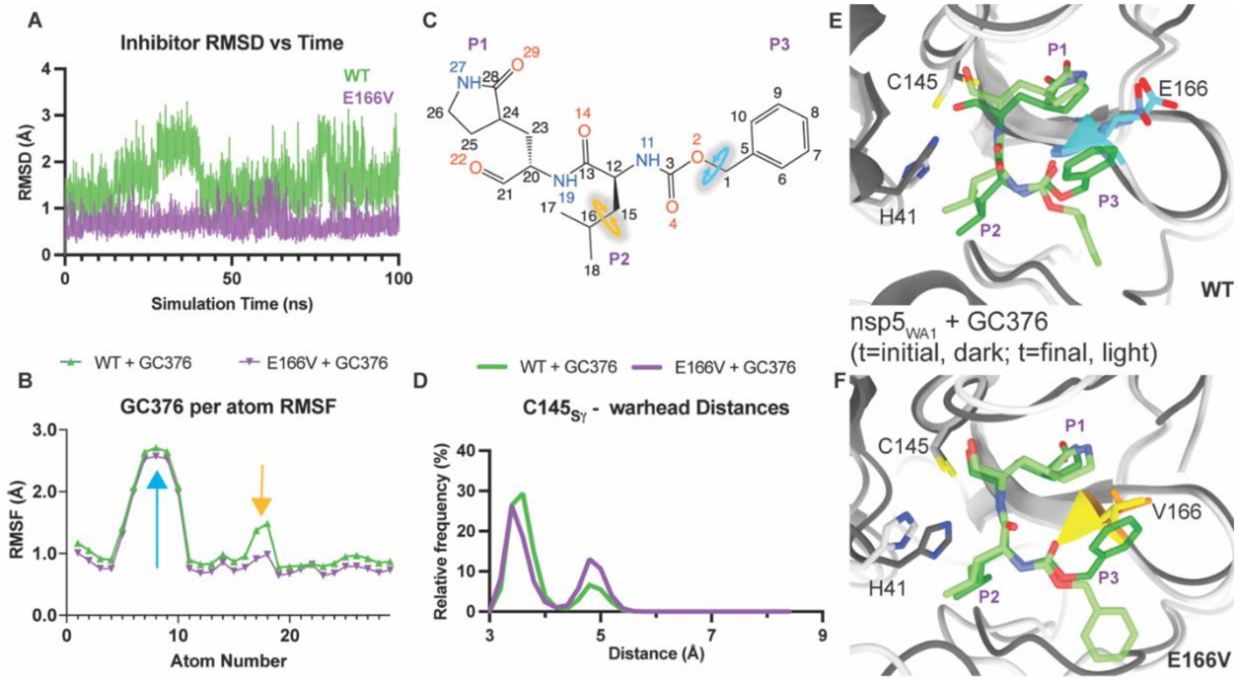

Fig. S8. Structural adaptation of GC376 within the WT-$nsp5_{WA1}$ and E166V-$nsp5_{WA1}$ active sites. (A) RMSD of GC376 binding in the WT- and E166V-$nsp5_{WA1}$:GC376 complexes, computed by aligning at every time point to the reference $nsp5_{WA1}$ backbone structure at the beginning of the simulation, then computing the RMSD of non-hydrogen GC376 atoms. The WT- and E166V-$nsp5_{WA1}$:GC376 simulations are in green and purple. **(B)** Positional RMSF of GC376 atoms, numbered as in (C). Blue and yellow arrows indicate the relatively mobile P2 and P3 groups shown in (C). These values represent the internal atomic fluctuations of GC376 at the end of the simulation compared to their starting positions. **(C)** 2D representation of GC376. Atom numbers correspond to the positions plotted on the horizontal axis in (B). Inhibitor sites are in purple, as previously defined. Rotating bonds corresponding to the colored arrows in (B) are indicated by circular arrows of the same color. **(D)** Frequency distribution (in percentage of total simulation time) of interatomic distances of the catalytic $C145_{S\gamma}$ in relation to the reactive aldehyde carbon in GC376 [C21 in (C)]. **(E, F)** Superimposition of WT-$nsp5_{WA1}$:GC376 (E) and E166V-$nsp5_{WA1}$:GC376 (F) complexes at the beginning and end of the simulation (dark *vs.* light colors, respectively; E166 is in blue, V166 in yellow, rest of nsp5 in gray, NIR in green). Catalytic residues H41 and C145 are labeled.

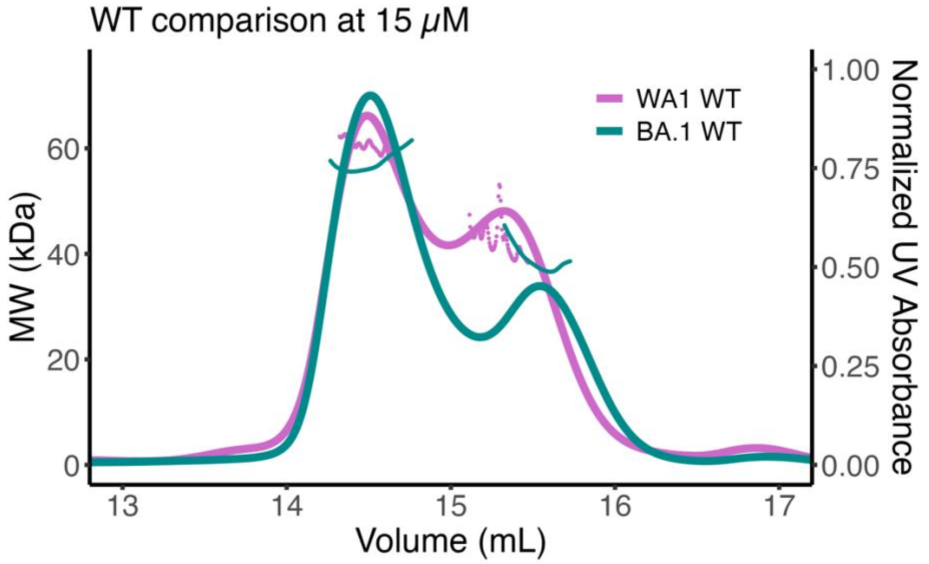

Fig. S9. Comparison of SEC-MALS elution profiles of WT-nsp5$_{WA1}$ and WT-nsp5$_{BA.1}$ at 15 μM. Points indicate the molecular weight (MW) calculated based on the light scattering and UV absorbance (normalized UV absorbance shown as a trace).

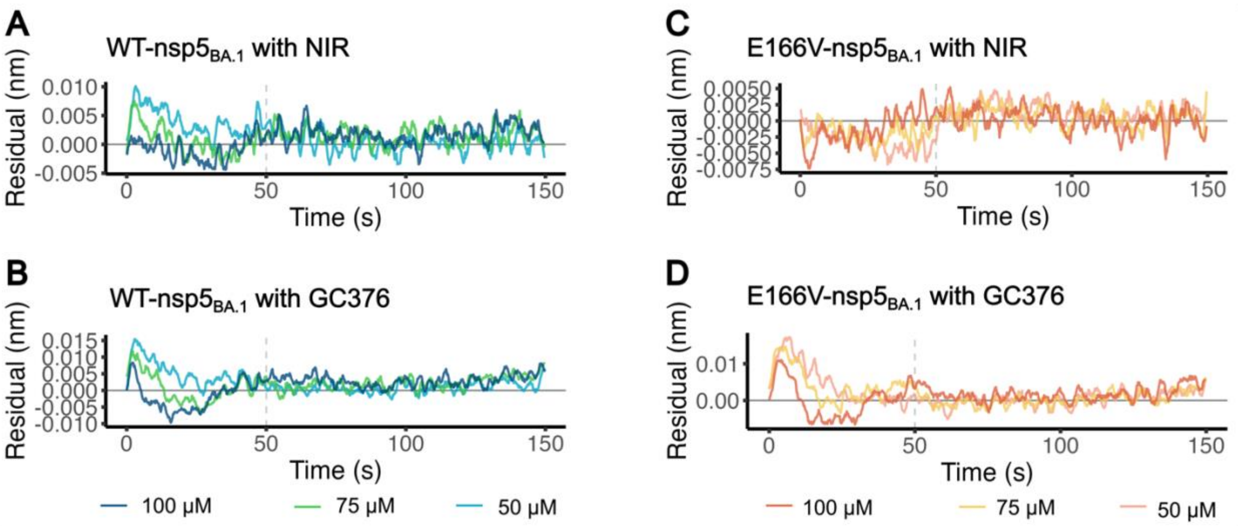

Fig. S10. Residuals of BLI fitting for WT-nsp5BA.1 **(A-B)** and E166V-nsp5BA.1 **(C-D)** with NIR or GC376 as indicated.

Supplementary Tables

Table S1. IC_{50} values of WT- and E166V-nsp5 proteins. Values represent average and standard deviations from n = 3 replicates.

	**IC_{50} ± SD / µM (Fold Change from WT_{WA1})**	
**$nsp5_{WA1}$ enzyme**	**NIR**	**GC376**
WT-$nsp5_{WA1}$	1.39 ± 0.7 **(1)**	1.67 ± 0.9 **(1)**
E166V-$nsp5_{WA1}$	12.5 ± 9 **(9)**	1.12 ± 0.3 **(0.7)**
	**IC_{50} ± SD / µM (Fold Change from $WT_{BA.1}$)**	
**$nsp5_{BA.1}$ enzyme**	**NIR**	**GC376**
WT-$nsp5_{BA.1}$	0.778 ± 0.06 **(1)**	1.13 ± 0.1 **(1)**
E166V-$nsp5_{BA.1}$	26.5 ± 18 **(34)**	1.27 ± 1.6 **(1.3)**

Table S2. Inflection temperatures (T_i) for $nsp5_{WA1}$ and $nsp5_{BA.1}$ recombinant proteins with DMSO, NIR, or GC376. dT_i denotes the difference between the respective drug complex and the DMSO control. Values represent average and standard deviations from n = 3 replicates.

	T_i ± SD / °C (dT_i from DMSO)		
**$nsp5_{WA1}$ enzyme**	**DMSO**	**NIR**	**GC376**
WT-$nsp5_{WA1}$	56.3 ± 2.1	76.0 ± 0.8 **(+ 19.6)**	77.4 ± 0.2 **(+ 21.1)**
E166V-$nsp5_{WA1}$	57.9 ± 0.7	59.0 ± 1.3 **(+ 1.1)**	68.8 ± 0.3 **(+ 10.9)**
WT-$nsp5_{BA.1}$	58.4 ± 0.1	75.6 ± 4.1 **(+ 17.2)**	78.0 ± 0.3 **(+ 19.6)**
E166V-$nsp5_{BA.1}$	57.4 ± 0.1	59.2 ± 0.3 **(+ 1.8)**	68.0 ± 0.5 **(+ 10.7)**

Table S3. Calculated molecular weight (MW) and percent mass (Mass %) from SEC-MALS. The MW and Mass % of each monomer and dimer peaks. Errors given as a percentage of the MW.

	Dimer peak		Monomer peak	
	**MW / kDa**	**Mass %**	**MW / kDa**	**Mass %**
**WT-nsp5$_{WA1}$ (60 µM)**	67.9 (± < 0.1%)	100	N/A	N/A
**WT-nsp5$_{WA1}$ (15 µM)**	61.5 (± 0.2%)	55.7	42.2 (± 0.3%)	44.3
**WT-nsp5$_{WA1}$ + NIR**	69.5 (± 0.1%)	100	N/A	N/A
**WT-nsp5$_{WA1}$ + GC**	71.7 (± 0.1%)	100	N/A	N/A
**WT-nsp5$_{BA.1}$ (60 µM)**	63.2 (± < 0.1%)	71.7	36.4 (± 0.2%)	28.3
**WT-nsp5$_{BA.1}$ (15 µM)**	57.3 (± 0.1%)	70.5	38.9 (± 0.3%)	29.5
**WT-nsp5$_{BA.1}$ + NIR**	67.8 (± 0.1%)	100	N/A	N/A
**WT-nsp5$_{BA.1}$ + GC**	68.3 (± 0.1%)	100	N/A	N/A
**E166V-nsp5$_{WA1}$ (60 µM)**	N/A	N/A	40.0 (± 0.1%)	100
**E166V-nsp5$_{WA1}$ (15 µM)**	N/A	N/A	36.2 (± 0.1%)	100
**E166V-nsp5$_{WA1}$ + NIR**	N/A	N/A	38.2 (± 0.2%)	100
**E166V-nsp5$_{WA1}$ + GC**	64.3 (± 0.1%)	89.8	41.1 (± 0.4%)	10.2
**E166V-nsp5$_{BA.1}$ (60 µM)**	N/A	N/A	43.1 (± 0.2%)	100
**E166V-nsp5$_{BA.1}$ (15 µM)**	N/A	N/A	36.4 (± 0.3%)	100
**E166V-nsp5$_{BA.1}$ + NIR**	N/A	N/A	37.7 (± 0.2%)	100
**E166V-nsp5$_{BA.1}$ + GC**	64.3 (± 0.1%)	83.1	37.6 (± 0.3%)	16.9

Table S4. Summary of X-ray data collection and refinement statistics.

	E166V-nsp5$_{BA.1}$ + GC376	E166V-nsp5$_{BA.1}$ + NIR	E166V-nsp5$_{WA1}$ + GC376
Data collection			
X-ray source	APS 22-ID	APS 22-ID	NSLS-II 17-ID-2
Software	XDS	XDS	XDS
Space group	I2	I2	I2
Unit cell dimensions			
a, *b*, *c* (Å)	45.5, 53.8, 114.9	45.0, 53.2, 113.7	45.1, 53.5, 113.7
α, β, γ (°)	90.0, 100.9, 90.0	90.0, 101.1, 90.0	90.0,100.8, 90.0
ASU content	1	1	1
Wavelength (Å)	1.000000	1.000000	0.979338
Resolution range (Å)[a]	100.0–2.75 (2.91–2.75)	100.0–2.41 (2.55–2.41)	100.0–2.39 (2.53–2.39)
R_{merge}	0.093 (0.811)	0.036 (0.285)	0.10 (0.639)
R_{meas}	0.112 (0.969)	0.043 (0.338)	0.12 (0.758)
<I/σI>	8.6 (1.5)	18.02 (3.64)	9.08 (1.75)
$CC_{1/2}$ (%)	99.3 (74.0)	99.9 (96.4)	99.6 (65.0)
Completeness (%)	96.4 (92.5)	97.2 (96.3)	99.5 (98.3)
Redundancy	3.3 (3.2)	3.4 (3.4)	3.5 (3.4)
No. total reflections	22,867	34,061	37,007
No. unique reflections	6,951	10,034	10,643
Mosaicity	0.78	0.35	0.18
Refinement			
Resolution (Å)	48.6–2.76	55.9–2.41	34.1–2.39
No. test reflections[b]	322	520	558
R_{work} / R_{free}	0.207 / 0.265	0.206 / 0.256	0.193 / 0.229
No. atoms	2,395	2,362	2,505
Protein	2,359	2,327	2,403
Ligand/Ion	36	35	29
Water	0	0	73
Wilson B-factor (Å^2)	68.5	56.5	39.9
Average B-factors (Å^2)	76.0	68.0	41.0
RMS deviations			
Bond lengths (Å)	0.002	0.002	0.004
Bond angles (°)	0.44	0.62	0.70
MolProbity Statistics[c]			
All atom clash score	2.95	5.38	3.32
Rotamer outliers (%)	3.05	0.39	0.74
Cβ deviations>0.25 Å	0	0	0
Ramachandran[c]			
Favored region (%)	94.1	97.0	97.7
Allowed (%)	5.6	3.7	2.0
Outliers (%)	0.3	0.3	0.3
PDB accession code	9EEI	9EEV	9EET

[a] Values in parentheses are for the highest-resolution shell

[b] Random selection

[c] Values obtained from MolProbity
